# Applying Multivariate Segmentation Methods to Human Activity Recognition From Wearable Sensors’ Data

**DOI:** 10.2196/11201

**Published:** 2019-02-07

**Authors:** Kenan Li, Rima Habre, Huiyu Deng, Robert Urman, John Morrison, Frank D Gilliland, José Luis Ambite, Dimitris Stripelis, Yao-Yi Chiang, Yijun Lin, Alex AT Bui, Christine King, Anahita Hosseini, Eleanne Van Vliet, Majid Sarrafzadeh, Sandrah P Eckel

**Affiliations:** 1 Department of Preventive Medicine Keck School of Medicine of University of Southern California Los Angeles, CA United States; 2 Information Sciences Institute University of Southern California Los Angeles, CA United States; 3 Spatial Sciences Institute University of Southern California Los Angeles, CA United States; 4 Department of Radiological Sciences University of California Los Angeles Los Angeles, CA United States; 5 Department of Biomedical Engineering University of California, Irvine Irvine, CA United States; 6 Department of Computer Science University of California Los Angeles Los Angeles, CA United States

**Keywords:** machine learning, physical activity, smartphone, statistical data analysis wearable devices

## Abstract

**Background:**

Time-resolved quantification of physical activity can contribute to both personalized medicine and epidemiological research studies, for example, managing and identifying triggers of asthma exacerbations. A growing number of reportedly accurate machine learning algorithms for human activity recognition (HAR) have been developed using data from wearable devices (eg, smartwatch and smartphone). However, many HAR algorithms depend on fixed-size sampling windows that may poorly adapt to real-world conditions in which activity bouts are of unequal duration. A small sliding window can produce noisy predictions under stable conditions, whereas a large sliding window may miss brief bursts of intense activity.

**Objective:**

We aimed to create an HAR framework adapted to variable duration activity bouts by (1) detecting the change points of activity bouts in a multivariate time series and (2) predicting activity for each homogeneous window defined by these change points.

**Methods:**

We applied standard fixed-width sliding windows (4-6 different sizes) or greedy Gaussian segmentation (GGS) to identify break points in filtered triaxial accelerometer and gyroscope data. After standard feature engineering, we applied an Xgboost model to predict physical activity within each window and then converted windowed predictions to instantaneous predictions to facilitate comparison across segmentation methods. We applied these methods in 2 datasets: the *human activity recognition using smartphones* (*HARuS*) dataset where a total of 30 adults performed activities of approximately equal duration (approximately 20 seconds each) while wearing a waist-worn smartphone, and the Biomedical REAl-Time Health Evaluation for Pediatric Asthma (*BREATHE*) dataset where a total of 14 children performed 6 activities for approximately 10 min each while wearing a smartwatch. To mimic a real-world scenario, we generated artificial unequal activity bout durations in the BREATHE data by randomly subdividing each activity bout into 10 segments and randomly concatenating the 60 activity bouts. Each dataset was divided into ~90% training and ~10% holdout testing.

**Results:**

In the HARuS data, GGS produced the least noisy predictions of 6 physical activities and had the second highest accuracy rate of 91.06% (the highest accuracy rate was 91.79% for the sliding window of size 0.8 second). In the BREATHE data, GGS again produced the least noisy predictions and had the highest accuracy rate of 79.4% of predictions for 6 physical activities.

**Conclusions:**

In a scenario with variable duration activity bouts, GGS multivariate segmentation produced *smart-sized* windows with more stable predictions and a higher accuracy rate than traditional fixed-size sliding window approaches. Overall, accuracy was good in both datasets but, as expected, it was slightly lower in the more real-world study using wrist-worn smartwatches in children (BREATHE) than in the more tightly controlled study using waist-worn smartphones in adults (HARuS). We implemented GGS in an offline setting, but it could be adapted for real-time prediction with streaming data.

## Introduction

### Background

Time-resolved quantification of physical activity is important because physical activity is linked with human health. Physical activity has direct health benefits, and the American College of Sports Medicine and the Centers for Disease Control and Prevention [1] publish physical activity guidelines to promote and maintain public health (eg, children should do at least 60 min of physical activity per day). Physical activity also has indirect effects on health by modifying exposures of pollutants. The National Human Activity Pattern Survey [2] found that human activity patterns play a key role in explaining variation in pollutant exposures—by impacting the timing, location, and degree of exposures—and related health outcomes. It follows that high-resolution time-resolved monitoring of human activity may have clinical and research applications. Not only could a person’s moderate-to-vigorous activity (or inactivity) be logged to quantify typical spatio-temporal patterns but deviations from the typical routine could also be identified as possible targets for intervention. The widespread use of wearable smartphones and smartwatches, together with advances in communication, computation, and sensing capabilities, makes real-time human activity recognition (HAR) possible by providing remote data acquisition and on-device processing.

Indeed, wearable sensors and mobile devices are being increasingly used in studies assessing physical activity, sleep, mobility, medication adherence, and a variety of other areas [3]. Our study is motivated by the “Pediatric Research using Integrated Sensor Monitoring Systems” (PRISMS) program— launched in 2015 by the National Institute of Biomedical Imaging and Bioengineering—to develop a sensor-based, integrated health monitoring system for studying pediatric asthma. Asthma is a heterogeneous, multifactorial disease that is one of the most common causes of emergency hospital visits in children [4]. Important risk factors for asthma exacerbation include allergen and air pollutant exposures and viral infection [4], but physical activity also plays an important role in asthma incidence [5], acute symptoms [6], and long-term control [7,8]. In a framework such as PRISMS, HAR may facilitate the management of asthma and the identification of triggers of exacerbation.

### Windowing in Human Activity Recognition Modeling Approaches

Data for HAR are increasingly collected using wearable sensors (eg, accelerometers and gyroscopes) that permit continuous, real-time monitoring [9-13]. Most HAR studies summarize accelerometer and gyroscope data streams—as well as the resulting instantaneous activity predictions—using a time-based windowing approach. The reasons for this are two-fold. First, the typical duration of human activities is significantly longer than the sensors’ sampling rate (eg, 10-50 Hz). Second, raw data from an accelerometer or gyroscope are highly variable, noisy, and oscillatory, so instantaneous raw values may provide insufﬁcient information to differentiate the associated activity. The size of the window is constrained by the sensor sampling frequency and is an important parameter that affects the accuracy of the HAR prediction, the computational loads of the algorithm, and the energy consumption on the wearable device. When selecting the size of a fixed-size window, there is a trade-off between being too short (captures fine details and produces noisy predictions) and being too long (misses short-duration activity bouts and produces more stable predictions). In a platform such as PRISMS where researchers might want to tailor context-sensitive interactions with study participants (eg, triggering a notification or survey) based on physical activity patterns, windows that are too short could generate frequent interactions with users, leading to notification fatigue and reduced compliance. Longer windows could perform well at certain times of the day when activities are fairly constant over long periods (eg, sedentary classroom time) but poorly during periods of high variability (eg, gym class and getting ready for school). A variable-sized sampling window approach with data-driven break points (at times when the activities may change) has the potential to improve HAR and improve the usability of platforms involving HAR.

### Time Series Segmentation

Fixed-size sliding windows are 1 type of a larger class of segmentation methods in time series analysis. Segmentation methods divide a time series into segments having similar characteristics. Most segmentation algorithms can be framed in several ways: (1) producing the best representation using only a given number of segments, (2) producing the best representation such that the maximum error for any segment does not exceed the given threshold, or (3) producing the best representation such that the combined error of all segments is less than the given threshold [14]. Multivariate segmentation methods segment multidimensional signals. Multivariate segmentation has been studied in several contexts using various approaches (each with different assumptions), including Bayesian change point detection [15], hypothesis testing [16], mixture models, hidden Markov models [17], and convex segmentation [18]. For this study, we selected a multivariate segmentation algorithm called greedy Gaussian segmentation (GGS) [19], which is based on maximizing the likelihood of the data for a fixed number of segments. GGS assumes that in each segment, the mean and covariance are constant and independent of the means and covariances in all other segments. GGS is a scalable greedy algorithm and is applicable to solve much larger problems (in terms of vector dimension and time series length) than many of other above methods.

In this paper, we provide background on the GGS algorithm and perform a novel application of GGS to offline HAR, comparing GGS with the standard fixed-size sliding window approach. We use data from 2 HAR studies with different prescribed activity durations and different sensor wear modalities (waist-worn sensor and wrist-worn sensor). After processing the data using either segmentation approach, we used standard feature engineering and machine learning methods to predict activities and compared the accuracy of the 2 different segmentation approaches.

## Methods

### Data

The *human activity recognition using smartphones* (HARuS) dataset consists of 61 experiments conducted by 30 volunteers aged 19 to 48 years [20]. Triaxial accelerometery and gyroscope data were collected at 50 Hz by a waist-worn smartphone (Samsung Galaxy S II). Each experiment was about 7 min long. In each experiment, the HARuS protocol scripted 12 ambulation activities, including 6 basic activities (each approximately 20 seconds in duration) and 6 postural transition activities (stand-to-sit, sit-to-stand, sit-to-lie, lie-to-sit, stand-to-lie, and lie-to-stand). The 6 basic activities include 3 static postures (standing, sitting, and lying) and 3 dynamic activities (walking, walking downstairs, and walking upstairs). The raw data were directly acquired from the smartphone readings, and the activities were labeled by manual review of video recordings of each experiment. To be consistent with previous studies [11], we only modeled the 6 basic activities and deleted the 6 types of postural transition activity bouts and all unlabeled sessions, all of which were of relatively short duration and unlikely, for example, to be strongly associated with asthma exacerbation in studies using PRISMS [5]. The dataset was divided into the first 55 experiments for training (2 experiments each for 26 people and 3 experiments for 1 participant) and 6 experiments (2 experiments each for 3 people) for holdout testing. The 6 raw signals of experiment 1 are plotted in Multimedia Appendix 1.

The Los Angeles PRISMS Center *BREATHE* dataset [21-23] was collected on 16 participants, aged 5 to 15 years, using the BREATHE Kit, an informatics platform designed to monitor multiple exposures, behaviors, and activities in context to identify personal triggers and predict the risk of pediatric asthma exacerbations in real time. Triaxial accelerometry and gyroscope data were collected at 10 Hz using a wrist-worn Motorola Moto 360 Sport smartwatch. Participants performed each of the 5 activities (standing, sitting, lying, walking, and walking on stairs) for 10 min and running for 5 min (to minimize discomfort). Unlike the HARuS dataset, participants were permitted to perform natural movements (especially free arm movement such as sitting while typing or using a smartphone) during each activity. The raw data were acquired as the end product of a data pipeline (from smartwatch to the BREATHE app on the smartphone via Bluetooth and then securely uploaded to the BREATHE servers wirelessly and in real times). For the BREATHE dataset, we modeled all 6 scripted activities: standing, sitting, lying, walking, walking on stairs (labels did not differentiate up and down stairs), and running. We used experiments from 14 of the 16 participants as 2 participants had substantial quantities of missing data. In the BREATHE dataset, data were saved as separate files for each activity, for each participant. To evaluate whether GGS segmentation improves prediction under a scenario of variable activity bout durations, we generated artificial activity data files for each participant by (1) randomly dividing his or her activity sessions (each about 10 min long) into 10 subsessions; then (2) randomly shuffling all subsessions (60 in total); and finally (3) concatenating all 60 subsessions into 1 data file, potentially resulting in fewer than 60 distinct activity bouts if bouts with identical activities are located next to each other. Hence, we produced 14 artificial activity files with artificial unequal activity bout durations, one for each of the 14 participants. The artificial dataset was divided into the first 12 participants for training and the last 2 participants for holdout testing. The 6 raw signals of experiment 1 are plotted in Multimedia Appendix 1.

### Workflow

**Figure 1:**
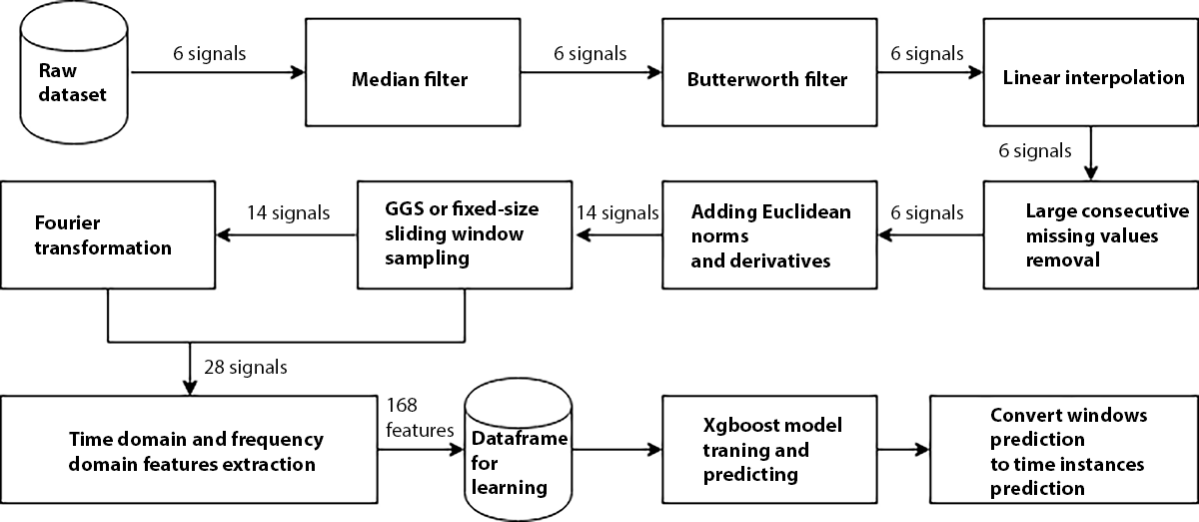
The workflow of the human activity recognition framework. GGS: greedy Gaussian segmentation.

Figure 1 provides an overview of our workflow. For both datasets, the raw data were first preprocessed by applying a median filter (kernel size=3) to remove outliers. Afterwards, a Butterworth [24] filter was used to remove artifacts and baseline wandering noise associated with the data acquisition process (eg, the constant force of gravity or shaking the device). Specifically, a third-order low-pass Butterworth filter was applied separately to each triaxial component (x, y, and z of the accelerometer and gyroscope). A power spectral density (PSD) was calculated and used to choose the cut-off frequency, over which the sensor signals were attenuated. PSD is a metric that estimates the distribution of power over frequency, and it has been widely implemented to evaluate filters of high-frequency with baseline-wandering noise [25].

Subsequently, the data streams were temporally aligned. The sampling frequency observed in practice can be a result of practical constraints (eg, battery saving and restricted access by the software stack in mobile device’s operating systems). Thus, observed data can be sampled irregularly, with mismatch between the 2 sensors. In the HARuS dataset, there were no mismatched time stamps (ie, only existing for 1 sensor) when we concatenated accelerometer and gyroscope readings according to their time stamps. However, the BREATHE dataset contained considerable mismatching, and both the accelerometer and the gyroscope were not perfectly collected at 10 Hz. To align the 2 sensor readings, we first downscale sampled the raw data at 50 Hz to round their time stamps to the nearest 50 Hz sampling point, and then we applied a linear interpolation method.

Specifically, we added (as necessary) records for all 50 Hz time stamps to both sensor data files and linearly interpolated missing sensor readings (approximately 80% because of the downscaling) based on the left 5 adjacent nonmissing values and the right 5 adjacent nonmissing values. In addition to the missing values caused by the mismatching time stamps, there was also a number of longer periods with missing values in the BREATHE dataset. After aligning the 2 sensors, we truncated time periods with more than 10 seconds of consecutive missing values.

Data transformation was used to augment the original data (6 signals from 2 triaxial sensors) with additional transformed signals. Statistical features were later extracted from both the raw and transformed signals. Specifically, 8 new signals were generated: 6 derivatives with respect to time (1 for each of the 6 original signals) and 2 Euclidean norms (1 for the x-, y-, and z-axis of each sensor). Hence, a total of 14 signals were available (6 original measured signals and 8 new calculated signals).

Time windows were generated using 2 approaches. First, multivariate segmentation on the 6 original signals produced windows of varying sizes, with break points selected using training data to reflect changes of the means and covariances of the raw signals (a detailed description follows). Second, for comparison, we created various sizes of nonoverlapping fixed-length sliding windows (4 sizes for HARuS dataset: 0.2 second, 0.8 second, 3 seconds, and 8 seconds; 6 sizes for BREATHE dataset: 0.2 second, 0.8 second, 3 seconds, 8 seconds, 12 seconds, and 40 seconds). Window sizes were chosen to include approximately the window size in the original HARuS study (2.56 seconds) [20] and to reflect a wide enough range to include the optimum window size for both datasets.

Within each set of windows, we extracted statistical features for input into a machine learning model. These statistical features were either based on time domain (the original time-based windows) or frequency domain (Fourier transformation of the original time-based windows). For each set of windows, we calculated a total of 168 features: 6 statistics (arithmetic mean, SD, median absolute deviation, minimum, maximum, and entropy) on 14 signals and on both the time and frequency domains (6 x 14 x 2=168).

### Multivariate Segmentation

A brief description of GGS [19] is as follows. Consider a multivariate time series consisting of *T* time instants *x*_*1*_, *x*_*2*_,..., *x*_*T*_ ∈ *R*^*m*^, where *m* is the number of features (ie, m=6 in our study). The time series need not be uniformly sampled in real time (see note in Discussion on the independence assumption). Given K break points *b*_*1*_,..., *b*_*K*_ ∈ (*1*,..., *T*) between a starting point *b*_*0*_= *1* and an end point *b*_*K+1*_= *T*, we assume that *x*_*t*_ ~ *MVN* (*µ*_*bi*_, *Σ*_*bi*_) ∀ *t* ∈ (*b*_*i*_,..., *b*_*i+1*_) ∀ *i* ∈ [*0*, *K*] and are independent samples, where *µ*_*bi*_ and *Σ*_*bi*_ denote the mean vector and covariance matrix of the multivariate normal distribution within the interval of (*b*_*i*_,.., *b*_*i+1*_). A GGS can be learned on the multivariate time series by fitting a greedy algorithm to maximize the covariance-regularized log-likelihood.

**Figure 2:**
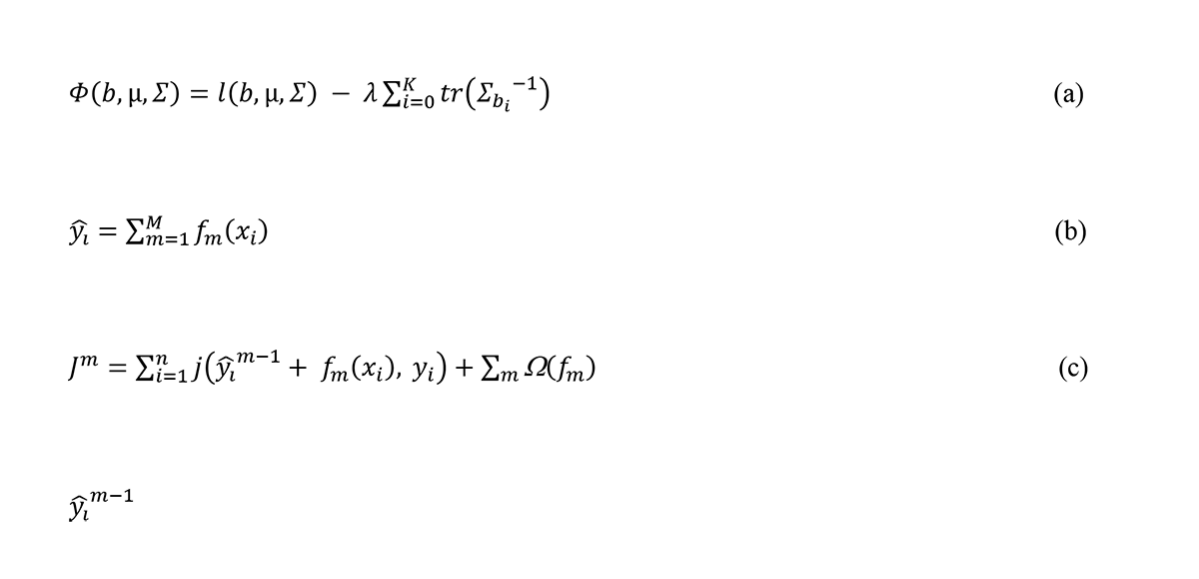
Equations.

In Figure 2 equation a, where *l* (*b*, *µ*, *Σ*) denotes the log-likelihood before regularization, *b* denotes the vector of break points, *µ* denotes [*µ*_*b0*_,..., *µ*_*bK*_], *Σ* denotes [*Σ*_*b0*_,..., *Σ*_*bK*_], and *λ ≥* 0 is an *a priori* specified hyperparameter that controls the amount of regularization [19]. The greedy heuristic algorithm follows a top-down subroutines of adding a new break point with the largest increase of *Φ(b,µ,Σ)* at each step until K, and then in a bottom-up way adjusts the positions of all break points until no change of any 1 break point increases *Φ(b,µ,Σ)*. A curve of the covariance-regularized log-likelihood versus K can be used to select K for a given dataset.

### Gradient Boosted Trees Classification

To achieve high accuracy using a scalable method, we predicted activity classes using Xgboost [26], an implementation of a tree-based boosting widely used in machine learning challenges. For a given dataset (D) with *n* observations and *p* features (ie, *p*=168 in our analysis), *D*={(*x*_*i*_ ∈ *R*^*p*^, *y*_*i*_ ∈ *R*)} ∀ *i* ∈[*1*, *n*], Xgboost ensembles M trees denoted *f*_*m*_ to predict the output *y*_*i*_.

The model is trained in a greedy, additive manner starting from m=1 (Figure 2, equation b). Let ŷ_i_^m^^−^^1^ be the prediction of y_i_ at the (m−1)^th^ iteration. We add f_m_ to minimize the following objective (J^m^) until the satisfying convergence between the prediction and the ground truth, where j is a predefined differentiable convex loss function that measures the difference between the current prediction and the ground truth and Ω is a predefined regularization term that penalizes the complexity of the model to prevent overfitting:

Xgboost has features that can outperform other implementations of tree-based boosting (eg, boosted trees in scikit-learn and generalized boosted regression model in R) such as (1) using an exact (or approximate, for large datasets) greedy algorithm to enumerate over all possible splits to find the best solution, (2) alleviating slow-downs using a cache-aware prefetching algorithm, and (3) enabling out-of-core computation by dividing the data into multiple blocks, each stored on disk, to use machine’s maximum resources (see Figure 2, equation c).

For the HARuS and BREATHE datasets, we tuned and implemented an Xgboost model with *m*=200 trees and learning rate=0.1 (more specifications in Tables 1 and 2) using *p*=168 features calculated on each segment (from fixed-sized windows or GGS) of the training data. Segment-specific predictions for the testing data were translated into instantaneous predictions to facilitate comparison across segmentation approaches. Final evaluations of accuracy were based on instantaneous predictions.

**Table 1:** Confusion matrix of instantaneous predictions using greedy Gaussian segmentation from the 6 test experiments in the Human Activity Recognition using Smartphones dataset.

True categories	Xgboost^a^ predicted categories						Recall (%)	Precision (%)
	W^b^	WU^c^	WD^d^	ST^e^	STD^f^	LY^g^		
W	11238	0	935	0	0	0	100	92.32
WU	0	11070	1297	0	12	0	99.61	89.43
WD	0	0	11659	0	40	0	83.36	99.66
ST	0	0	0	11037	2798	0	85.14	79.78
STD	0	24	96	1926	12546	0	81.49	85.98
LY	0	19	0	0	0	15266	100	99.88

^a^Xgboost specification: base_score=0.5, booster=“gbtree,” colsample_bylevel=1, colsample_bytree=1, gamma=0, learning_rate=0.1, max_delta_step=0, max_depth=2, min_child_weight=1, missing=None, n_estimators=200, n_jobs=1, nthread=None, objective=“multi:softprob,” random_state=0, reg_alpha=0, reg_lambda=1, scale_pos_weight=1, seed=None, silent=True, subsample=1. Overall accuracy: 91.06%.

^b^W: walking.

^c^WU: walking upstairs.

^d^WD: walking downstairs.

^e^ST: sitting.

^f^STD: standing.

^g^LY: laying.

**Table 2:** Confusion matrix of instantaneous predictions using greedy Gaussian segmentation from the 2 test experiments in the BREATHE dataset.

True categories	Xgboost^a^ Predicted categories						Recall (%)	Precision (%)
	L^b^	R^c^	ST^d^	STR^e^	STD^f^	WK^g^		
L	38874	166	6920	830	3938	0	76.63	68.76
R	1587	31593	0	12402	791	11693	54.41	82.54
S	12483	0	38596	864	8030	154	64.19	72.65
STR	559	6505	1929	46751	2320	6156	72.80	71.17
STD	887	0	5127	0	54300	77	89.91	72.05
WK	2146	12	555	4846	5976	52455	79.49	74.37

^a^Xgboost specification: base_score=0.5, booster=“gbtree,” colsample_bylevel=1, colsample_bytree=1, gamma=0, learning_rate=0.1, max_delta_step=0, max_depth=3, min_child_weight=1, missing=None, n_estimators=200, n_jobs=1, nthread=None, objective=“multi:softprob,” random_state=0, reg_alpha=0, reg_lambda=1, scale_pos_weight=1, seed=None, silent=True, subsample=1. Overall accuracy: 79.4%.

^b^L: lie.

^c^R: run.

^d^S: sit.

^e^STR: stair.

^f^STD: stand.

^g^WK: walk.

## Results

### Human Activity Recognition Using Smartphones Dataset

**Figure 3:**
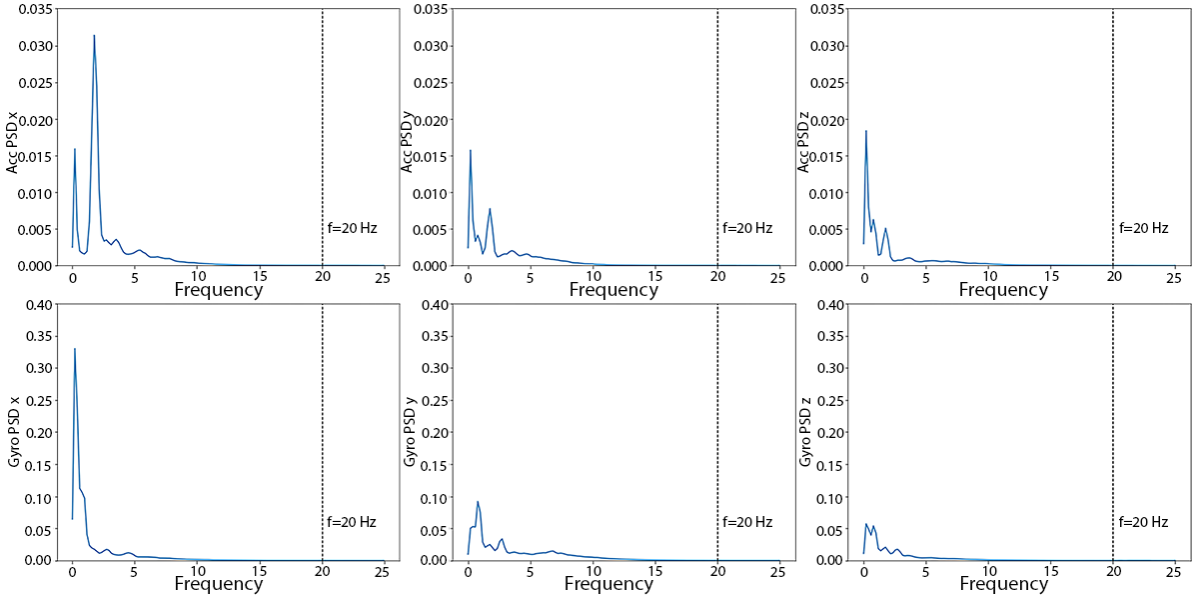
Triaxial (x, y, and z) power spectra density curves of accelerometer (top row) and gyroscope (bottom row) of the human activity recognition using smartphones training dataset. ACC: accelerometer; Gyro: gyroscope; PSD: power spectral density.

The PSD curves to determine the cut-off frequency of the Butterworth filter are displayed in Figure 3. All 6 PSD curves taper to 0 at higher frequencies, with largest values in the lower frequency range from 0 Hz to 5 Hz. There is little baseline wandering noise in high frequencies (>10 Hz). For consistency with previous studies [11], we chose 20 Hz as the cut-off frequency.

**Figure 4:**
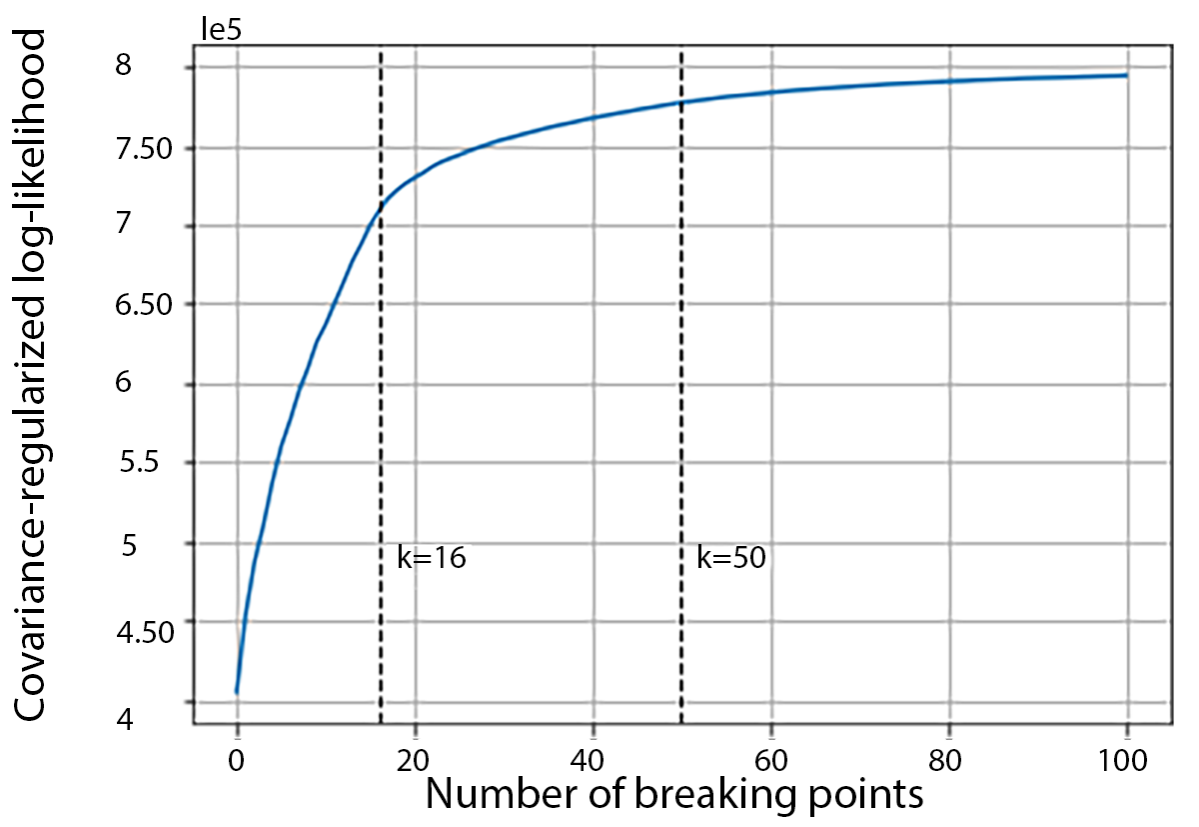
Total covariance-regularized log-likelihood curve of the human activity recognition using smartphones training dataset.

For GGS in the HARuS training data, the total covariance-regularized log-likelihood elevated rapidly as K increased from 0 to an inflection point around 16, and then even less rapidly (Figure 4). To favor more detailed segmentation results and allow for some incorrectly identified break points, especially during noisy periods and the transitory periods, we conservatively selected 50 break points.

**Figure 5:**
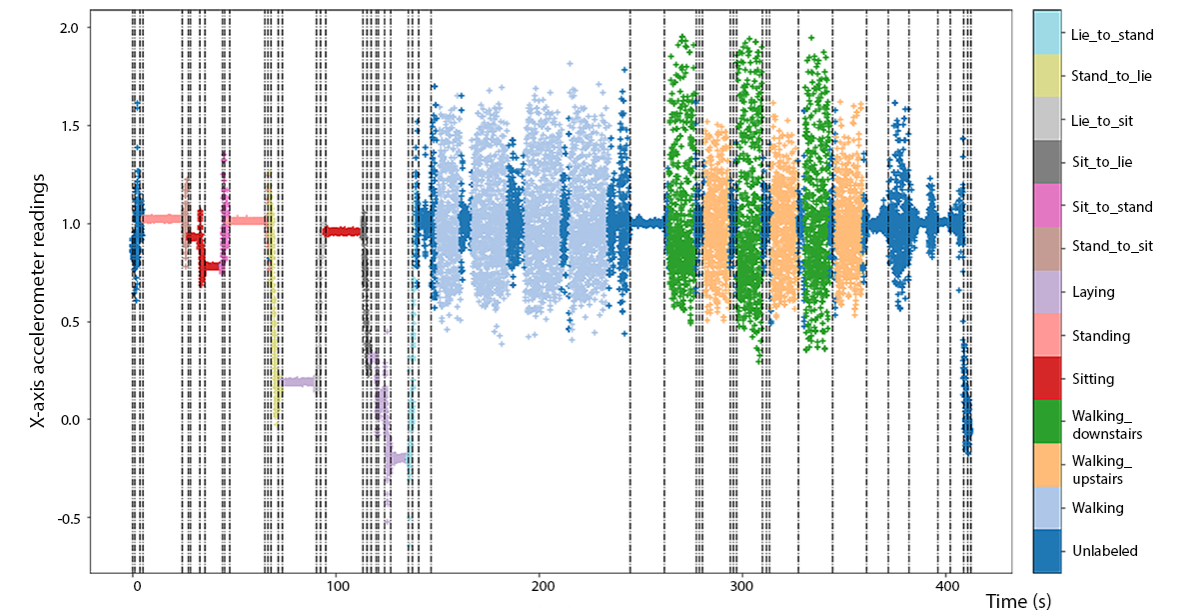
Multivariate segmentation break points (K=50) displayed using vertical dashed lines on the time series of x-axis accelerometer readings from experiment 1 in the human activity recognition using smartphones training dataset.

As shown in Figure 5 for experiment 1, the 13 bouts of the 6 nontransitory activities were generally well separated by the 50 break points. For this experiment, the first bout of sitting and the second bout of laying were both relatively noisy, and erroneous break points were created within these sessions.

**Figure 6:**
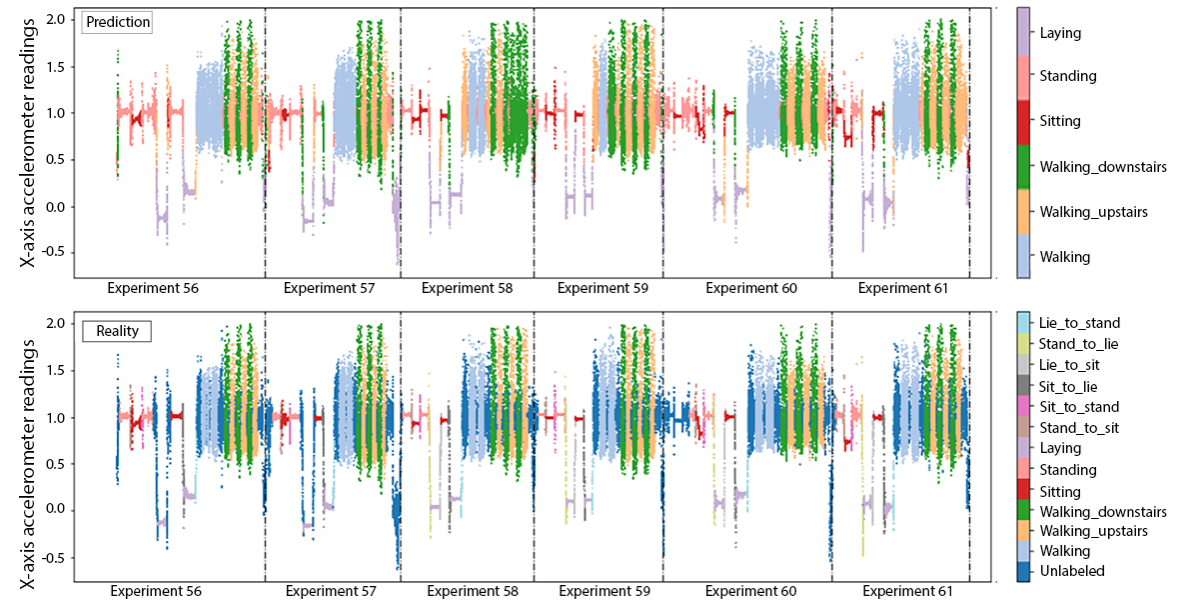
Instantaneous predictions using greedy Gaussian segmentation (top row) and ground truth (bottom row) from the 6 test experiments in the human activity recognition using smartphones dataset.

We trained an Xgboost model (Figure 6), a support vector machine (SVM) model using a radial basis function kernel and a random forest model using the segmented data. The instantaneous accuracy rate of the Xgboost model using GGS in the 6 holdout experiments was 91.06% (Table 1). This result is higher than the 89.3% accuracy reported in the original HARuS study on the same set of 6 activities [11], and it also should be noted that their accuracy was calculated using sliding window predictions and not instantaneous predictions. Had we calculated accuracy using segment-level predictions, our accuracy would have been 95.96%. When activities were misclassified, they tended to be misclassified as other similar energy activities (Table 1). For example, sitting was most frequently misclassified as standing. The results of the SVM model and the random forest model are summarized in Multimedia Appendix 1.

**Figure 7:**
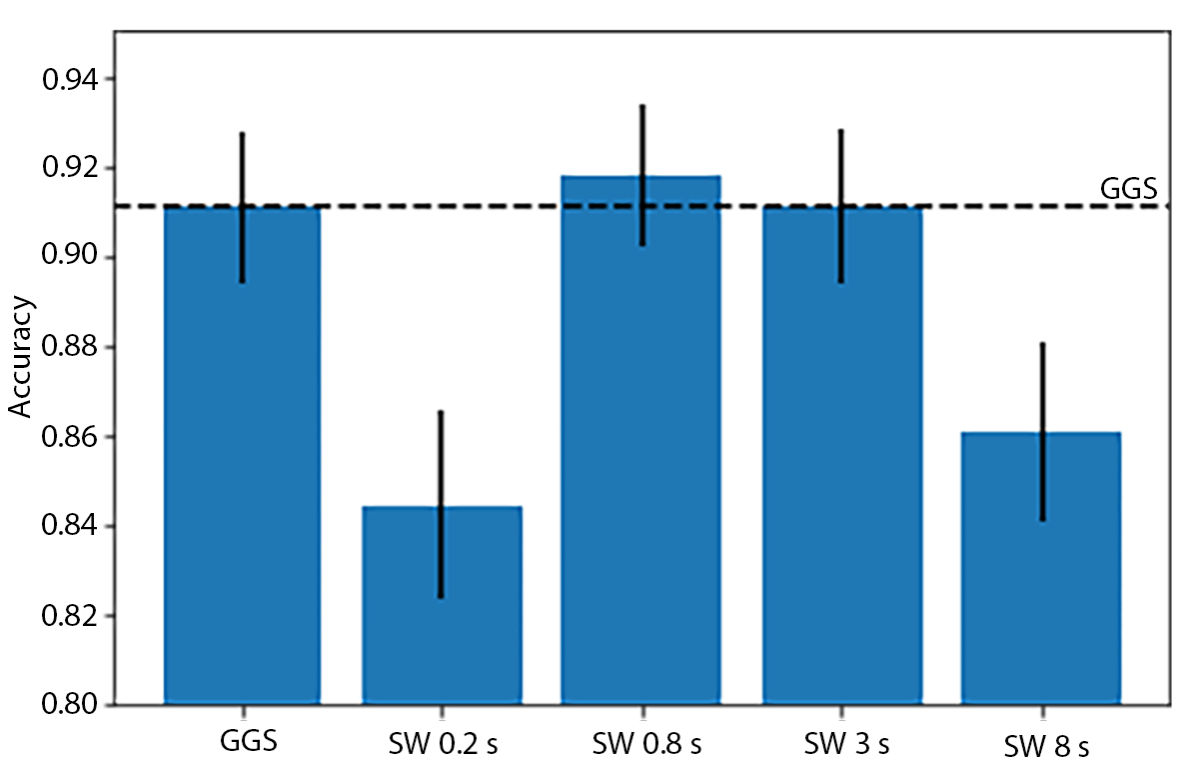
Accuracy of instantaneous predictions using 4 different fixed-size sliding windows (SWs) in the 6 test experiments in the human activity recognition using smartphones dataset. The horizontal dashed line represents the accuracy using greedy Gaussian segmentation. GGS: greedy Gaussian segmentation.

**Figure 8:**
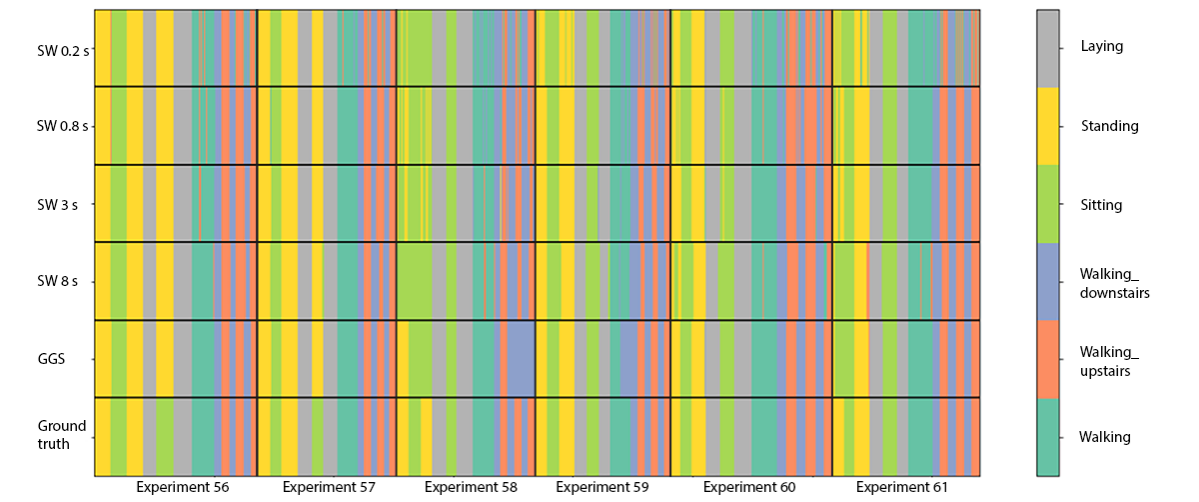
Predictions using 4 different fixed-sized sliding windows (SWs) and greedy Gaussian segmentation, as well as the ground truth for the 6 test experiments in the human activity recognition using smartphones dataset. GGS: greedy Gaussian segmentation; SW: sliding window.

**Figure 9:**
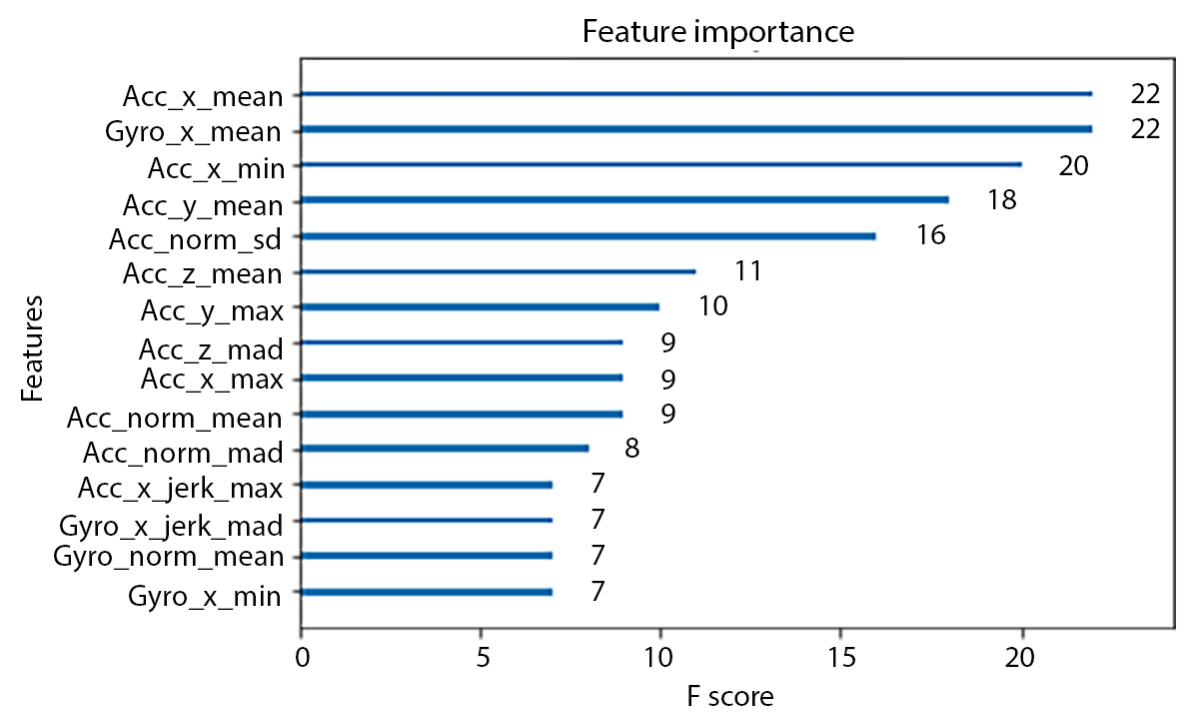
Importance of the top 15 features from Xgboost using greedy Gaussian segmentation from the human activity recognition using smartphones dataset. Abbreviations in the feature names are standard deviation (std), minimum (min), maximum (max), mean absolute deviation (mad), Euclidean magnitude (norm), and derivative (jerk). The operators in the names should be read in the order of from the right to the left. For example, acc_x_jerk_max means the maximum value of the derivative values on the x-axis of the accelerometer sensor. Acc: accelerometer; Gyro: gyroscope.

In comparison, the instantaneous accuracy of Xgboost models fitted using fixed-width sliding windows was highest for the 0.8-second window (91.79%), as shown in Figure 7. This *optimal* window size is smaller than the one used in the original HARuS paper (2.56 seconds) [20]. As might be expected from experiments designed to have equally sized activity bouts, the 0.8-second fixed-size sliding window accuracy was slightly higher than that from GGS (91.06%). In the HARuS data, predictions were relatively stable, with some additional variability for the smallest size sliding windows (Figure 8). The 3 most important features from Xgboost using GGS were the segment-specific mean, minimum of the x-axis of the accelerometer, and the mean of the x-axis of the gyroscope (Figure 9).

### BREATHE Dataset

**Figure 10:**
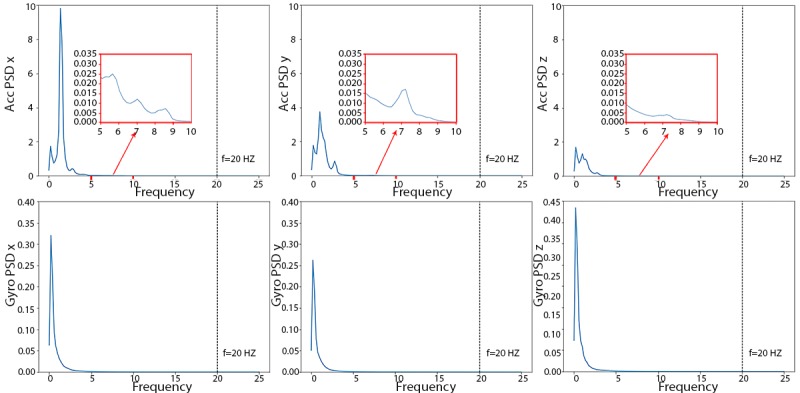
Triaxial (x, y, and z) power spectra density curves of accelerometer (upper 3 subplots) and gyroscope meter (lower 3 subplots) of the BREATHE training dataset. ACC: accelerometer; Gyro: gyroscope; PSD: power spectral density.

On the basis of the PSD plots of the training data (Figure 10), we again chose 20 Hz as the cut-off frequency for the Butterworth filter. The gyroscope energies are in the same scale as the HARuS dataset; however, the accelerometer readings have much larger amplitudes, which makes the curves look smoother in the range of approximately 5 Hz. The zoom-in windows in the accelerometer’s 3 subplots show the variations of the PSD curves in the range from 2.5 Hz to 7.5 Hz on a similar scale to that used in the PSD plots for the HARuS data.

**Figure 11:**
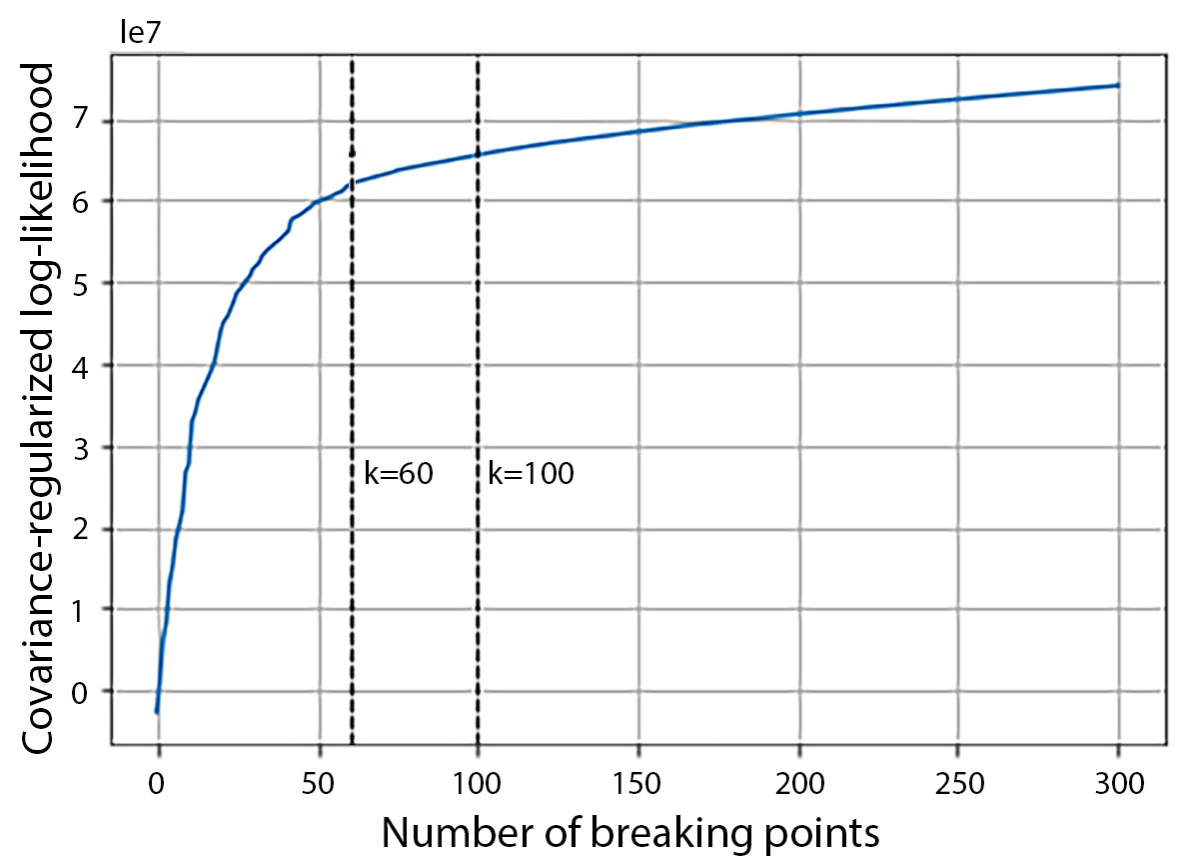
Total covariance-regularized log-likelihood curve of the BREATHE training dataset.

**Figure 12:**
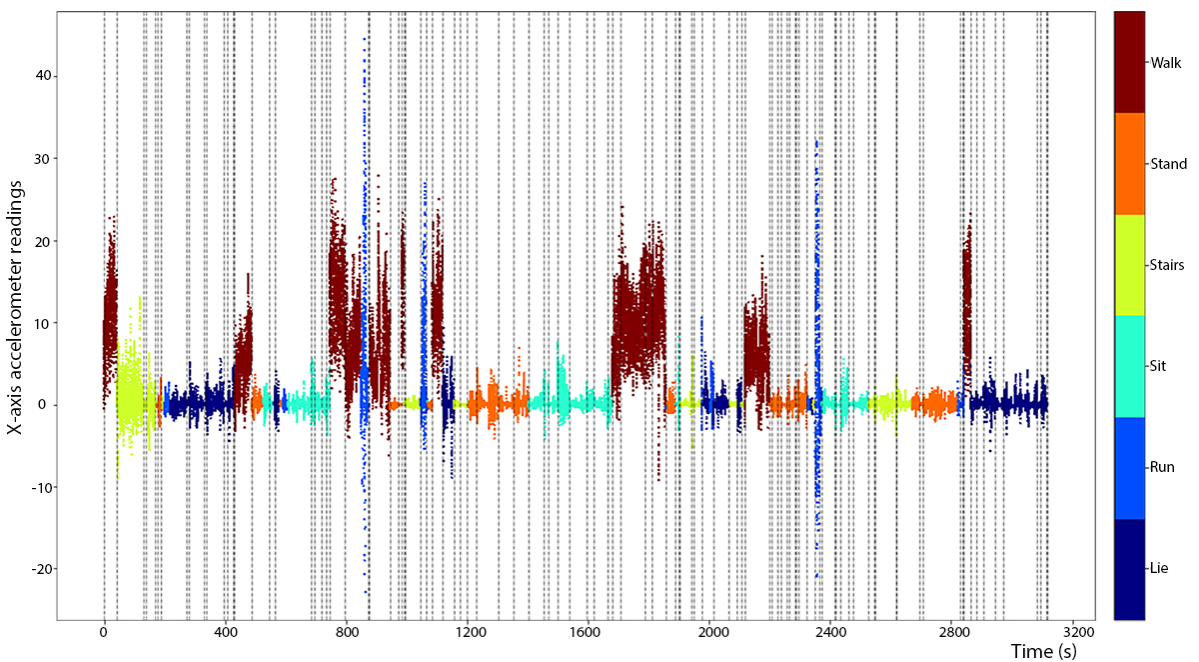
Multivariate segmentation break points (K=100) displayed using vertical dashed lines on the time series of x-axis accelerometer readings from experiment 1 in the BREATHE training dataset.

The covariance-regularized log-likelihood curve for the 12 training experiments in the BREATHE dataset (Figure 11) had one inflection point at approximately K=60 but no clear second inflection point (through K=300) as we had observed in the HARuS dataset. Interestingly, there were, by design, approximately 60 activity bouts in each BREATHE experiment, demonstrating that GGS again identified the number of different activity bouts. We arbitrarily chose K=100 break points for multivariate segmentation as it was a round number larger than the most obvious inflection point. From Figure 12, it appears that 100 was an adequate number of break points. A choice of 60 break points would have been inadequate to segment approximately 60 bouts as some noisier bouts were erroneously partitioned into multiple segments.

**Figure 13:**
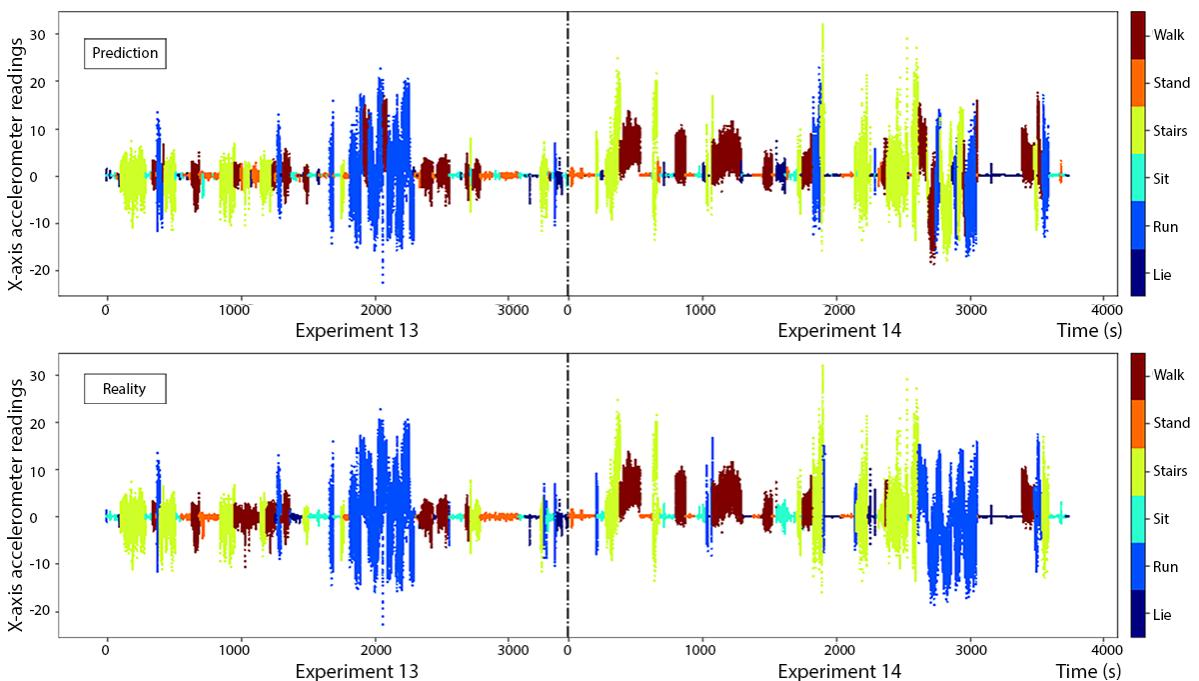
Instantaneous predictions using greedy Gaussian segmentation (top) and ground truth (bottom) from the 2 test experiments (13 and 14) in the BREATHE dataset.

**Figure 14:**
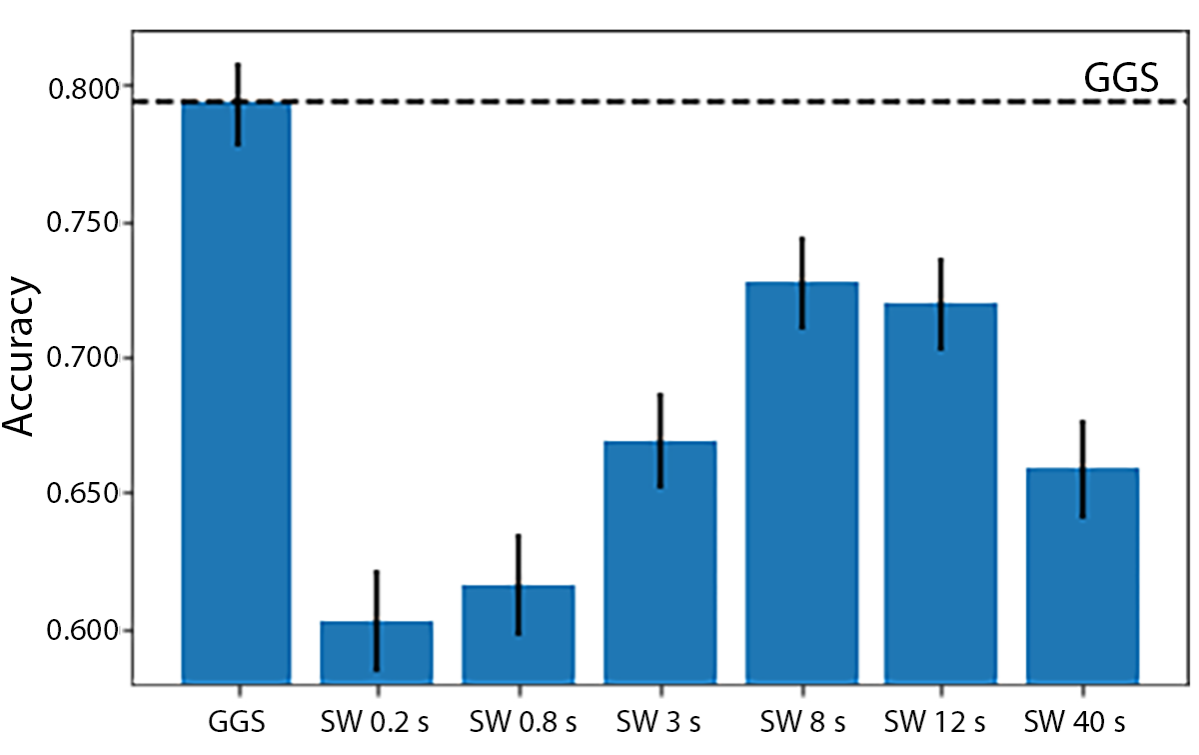
Accuracy of instantaneous predictions from Xgboost using 6 different fixed-size sliding windows (SWs) in the 2 test experiments in the BREATHE dataset. The horizontal dashed line represents the accuracy from Xgboost with greedy Gaussian segmentation. SW: sliding window; GGS: greedy Gaussian segmentation.

Similar to the HARuS dataset, 3 models were trained: Xgboost, SVM, and random forestAs evident from Figure 13, the predictive accuracy for certain activities varied across participants (eg, the accuracy for running was 71.5% for the participant in experiment 13 and 74.4% for the participant in experiment 14). Similar to the HARuS results, most misclassified records were shuffled either within the active group (walk, stair, and run) or the inactive group (sit, lie, and stand). If the activities had been grouped into active or inactive, the instantaneous accuracy rate would have been 95.0%. The results of the SVM model and the random forest model are shown in Multimedia Appendix 1. The instantaneous accuracy rate of the Xgboost model using GGS was 79.4% (Table 2 and Figure 14).

**Figure 15:**
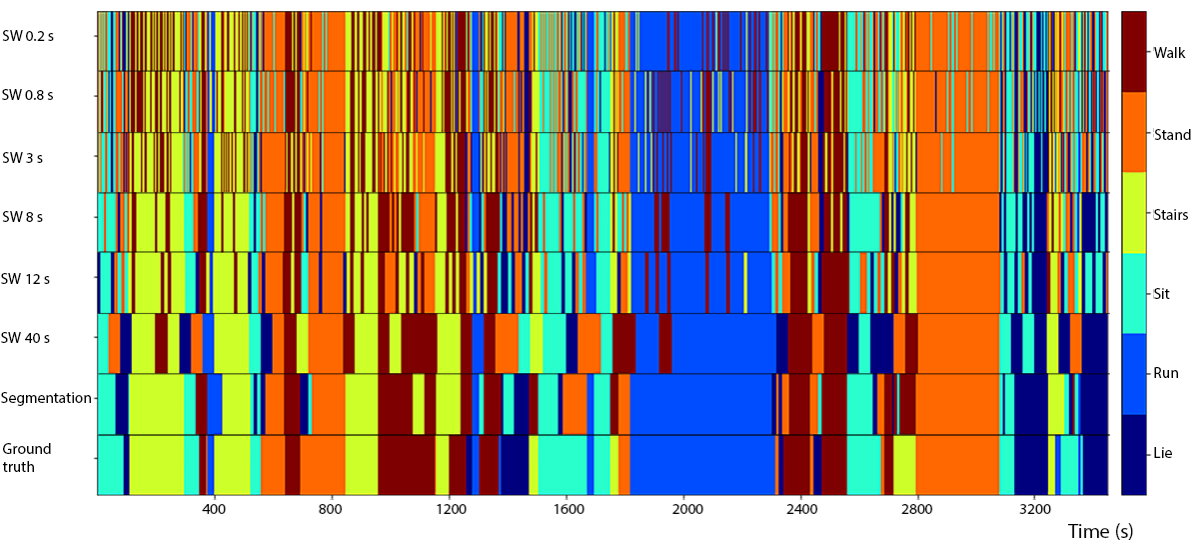
Predictions from Xgboost using 6 different fixed-sized sliding windows (SWs) and greedy Gaussian segmentation as well as the ground truth for experiment 13 of the BREATHE test data. SW: sliding window; GGS: greedy Gaussian segmentation.

**Figure 16:**
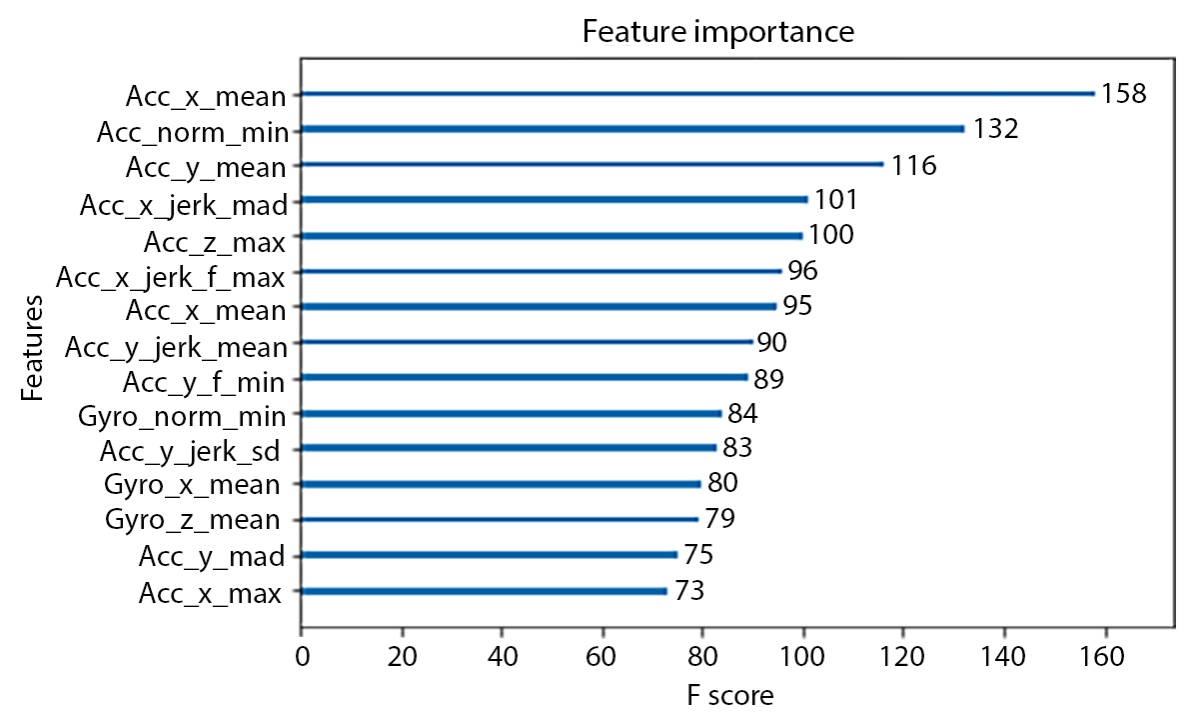
Importance of the top 15 features from Xgboost using greedy Gaussian segmentation from the BREATHE dataset. Abbreviations in the feature names are SD, minimum (min), maximum (max), mean absolute deviation (mad), Euclidean magnitude (norm), and derivative (jerk). The operators in the names should be read in the order of from the right to the left. For example, acc_x_jerk_max means the maximum value of the derivative values on the x-axis of the accelerometer sensor.

The accuracies of Xgboost from the 4 smallest fixed-size sliding windows (the same sizes as used in the HARuS dataset) increased monotonously. To achieve the reverse U-shape curve indicating that we obtained the optimum window size, we included 2 additional window sizes. The highest accuracy was achieved for the 8-second window (72.7%) as shown in Figure 13. As expected in this dataset with activity bouts of unequal duration, the *smart-sized* GGS segmentation (79.4% accuracy) considerably outperformed the fixed-size sliding windows. Not only was GGS more accurate but it also produced considerably less noisy predictions as shown in Figure 15. The 2 most important features from Xgboost using GGS were segment specific: mean z-axis and the minimum norm of the triaxial accelerometer signal (Figure 16).

## Discussion

### Summary of Findings

We found that Xgboost using GGS outperformed Xgboost using fixed-size sliding windows in a dataset with unequal activity bout durations (BREATHE), by producing more accurate and considerably more stable predictions. When implemented in a platform such as PRISMS, GGS should be able to identify short bursts of activity while still producing relatively smooth predictions. Identification of short activity bouts is particularly important for appropriately quantifying vigorous activity in children [27]. Noisy predictions from fixed-size sliding windows might need to be smoothed by pooling (ie, majority vote) for improved face validity of reported activity classifications and to avoid triggering excessive user notifications. Note that we presented our results using instantaneous predictions—to allow for comparisons across segmentation methods—that resulted in slightly lower accuracy than previous studies presenting segment-level predictions. In practice, segment-level predictions are typically used.

Major differences between the HARuS and BREATHE datasets included not only activity bout duration (equal vs unequal), participant ages (adults vs children), and experimental protocol (tightly proscribed activities vs activities allowing for more natural movements) but also how the sensors were worn. This difference in wear location is likely the cause of the differences between the most important features in the Xgboost models. The axes of a device (smartwatch or smartphone) are typically labeled as x, denoting the side-to-side dimension; y, denoting the forward and backward dimension; and z, denoting the up and down dimension. Incorporating these axes with the wearing position of the 2 datasets, forward movement would correspond to signal along the x-axis for HARuS participants and the z-axis (slightly deviated to x-axis) for BREATHE participants. For both datasets, the most important features appeared to be related to forward motion (x-axis for the HARuS data and z-axis or combination of axes, ie, the norm for the BREATHE data) and the direction perpendicular to this motion (eg, mean values of the y-axis of the accelerometer, acc_y_mean, which had the third highest score in the HARuS data and the fourth highest score in the BREATHE data).

### Limitations

In this study, the models were trained by *clip-independent* method. Time dependency is more obvious in datasets with temporal context, and many researches applied hidden Markov model (HMM) to such datasets as motion videos or images [28], body makers [29], and so on. For pure waist- or wrist-worn accelerometer or gyroscope meter, the signals do not have the strong time dependency as those temporal context data. Second, to compare the *time-dependent* methods, HMM should be tested with other analogic methods such as long short-term memory (LSTM), but not GGS. GGS is a way to clip the data such as the *fixed-length* sliding window. We can either apply *clip-independent* method as in this study or HMM or LSTM to test the time dependency among those clips.

The major weaknesses of the GGS approach are computational load and space requirements. To deploy GGS on streaming data, we would need to maintain a much larger cache memory of the latest received streaming data in comparison with the traditional fixed-length sliding window methods. GGS also requires time series of continuous features. However, sensor data (such as accelerometer and gyroscope) are typically quantitative, so this requirement is reasonable. Furthermore, missing values need to be either removed or interpolated. As for scalability, GGS has a runtime complexity of O(KTn^3^) in the normal mode and O(Tn^3^) in a *warm start* mode, in which the algorithm directly starts with a random set of K breaking points. Fixed-size sliding window approaches have better runtime complexity of O(n). Thus, the greedy heuristics needs to be improved in our future study. However, as the number of segments (K) is generally much smaller than the optimum number of fixed-size windows, GGS could largely save computational loads in the subsequent feature engineering, especially when tremendous feature to be extracted. Statistically, the GGS algorithm assumes that the multivariate time series can be described as independent samples from a multivariate Gaussian distribution within each segment. Time series data typically display autocorrelation, which would violate the independence assumption, especially when breaking points were not enough to separate the autocorrelated parts into different segments.

### Conclusions

Identification of the break points that signify changes in physical activity plays an important role in quantifying HAR. In platforms such as PRISMS, HAR can be used not only to quantify the total duration of time in, for example, light, moderate, or vigorous activity but also to trigger user notifications or alerts or provide real-time feedback on activity. Our GGS-based approach shows great potential in variable activity bout duration scenarios and produces fewer variable predictions that should minimize unnecessary interactions with the user. However, computational and implementation limitations exist. Interesting future work will be focused on deploying GGS in real-time data streams and, more generally, finding heterogeneous segments when introducing additional sensor signals measured at different frequencies and on different scales (eg, sensors for physiological signals such as heart rate).

## Appendix

Multimedia Appendix 1 Supplemental tables and figures.

## Abbreviations

GGS: greedy Gaussian segmentation
HAR: human activity recognition
HARuS: human activity recognition using smartphones
HMM: hidden Markov model
LSTM: long short-term memory
PRISMS: Pediatric Research using Integrated Sensor Monitoring Systems
PSD: power spectral density
SVM: support vector machine
