# Supplementary material for: Applying Multivariate Segmentation Methods to Human Activity Recognition From Wearable Sensors’ Data
Source: JMIR Mhealth Uhealth. 2019 Feb 7;7(2):e11201. doi: 10.2196/11201 (PMC6386646; doi:10.2196/11201)
Supplement: Multimedia Appendix 1 [file mhealth_v7i2e11201_app1.pdf]

Appendix

Table 1A: Confusion matrix of instantaneous predictions using GGS from the 6 test experiments in the HARuS dataset (Random Forest)

True Categories	Predicted Categories						Recall	Precision
	WALKING	WALKING UPSTAIRS	WALKING DOWNSTAIRS	SITTING	STANDING	LAYING		
**WALKING**	12173	0	0	0	0	0	73.21%	100%
**WALKING UPSTAIRS**	3851	6022	2465	0	41	0	100%	48.64%
**WALKING DOWNSTAIRS**	602	0	11057	0	40	0	81.77%	94.51%
**SITTING**	0	0	0	7655	6179	1	57.37%	55.33%
**STANDING**	0	0	0	5687	8905	0	58.72%	61.02%
**LAYING**	0	0	0	0	0	15285	99.99%	100%
Random Forest Specification: bootstrap=True, class_weight='balanced', criterion='gini', max_depth=2, max_features='auto', max_leaf_nodes=None, min_impurity_decrease=0.0, min_impurity_split=None, min_samples_leaf=1, min_samples_split=2, min_weight_fraction_leaf=0.0, n_estimators=10, n_jobs=1, oob_score=False, random_state=None, verbose=0, warm_start=False<br>Overall accuracy: **76.40%**								

Table 2A: Confusion matrix of instantaneous predictions using GGS from the 6 test experiments in the HARuS dataset (SVM)

True Categories	Predicted Categories						Recall	Precision
	WALKING	WALKING UPSTAIRS	WALKING DOWNSTAIRS	SITTING	STANDING	LAYING		
**WALKING**	12173	0	0	0	0	0	100%	100%
**WALKING UPSTAIRS**	0	12350	29	0	0	0	55.94%	99.76%
**WALKING DOWNSTAIRS**	0	9252	2433	0	14	0	72.28%	20.79%
**SITTING**	0	166	1	13585	29	54	92.99%	98.19%
**STANDING**	0	246	903	1024	12419	0	82.89%	85.10%
**LAYING**	0	62	0	0	2519	12704	99.57%	83.11%
SVM Specification: C=1.0, cache_size=200, class_weight=None, coef0=0.0, decision_function_shape='ovr', degree=3, gamma='auto', kernel='rbf', max_iter=-1, probability=False, random_state=None, shrinking=True, tol=0.001, verbose=False<br>Overall accuracy: **82.12%**								

Table 3A: Confusion matrix of instantaneous predictions using GGS from the 6 test experiments in the BREATHE dataset (Random Forest)

True Categories	Predicted Categories						Recall	Precision
	WALKING	WALKING UPSTAIRS	WALKING DOWNSTAIRS	SITTING	STANDING	LAYING		
**WALKING**	38533	0	448	1326	8682	1739	65.72%	75.96%
**WALKING UPSTAIRS**	24	44769	0	12046	1227	0	57.11%	77.10%
**WALKING DOWNSTAIRS**	19075	101	13456	4586	22909	0	90.57%	22.37%
**SITTING**	111	29973	395	29439	2849	1453	28.00%	45.84%
**STANDING**	253	0	558	401	58281	898	60.37%	96.50%
**LAYING**	632	3546	0	57339	2591	1882	31.51%	28.51%
Random Forest Specification: bootstrap=True, class_weight='balanced', criterion='gini', max_depth=2, max_features='auto', max_leaf_nodes=None, min_impurity_decrease=0.0, min_impurity_split=None, min_samples_leaf=1, min_samples_split=2, min_weight_fraction_leaf=0.0, n_estimators=10, n_jobs=1, oob_score=False, random_state=None, verbose=0, warm_start=False<br>Overall accuracy: **51.84%**								

Table 4A: Confusion matrix of instantaneous predictions using GGS from the 6 test experiments in the BREATHE dataset (SVM)

True Categories	Predicted Categories						Recall	Precision
	WALKING	WALKING UPSTAIRS	WALKING DOWNSTAIRS	SITTING	STANDING	LAYING		
**WALKING**	24242	3366	11742	2102	5200	4076	62.8%	47.78%
**WALKING UPSTAIRS**	1927	39297	5764	1602	1187	8289	35.66%	67.67%
**WALKING DOWNSTAIRS**	9842	1647	31999	3146	7603	5890	42.40%	53.21%
**SITTING**	1492	20564	13387	1453	2248	25076	15.88%	22.62%
**STANDING**	619	14039	9802	396	11628	23907	38.38%	19.25%
**LAYING**	478	31271	2759	447	2428	28607	29.84%	43.35%
SVM Specification: C=1.0, cache_size=200, class_weight=None, coef0=0.0, decision_function_shape='ovr', degree=3, gamma='auto', kernel='rbf', max_iter=-1, probability=False, random_state=None, shrinking=True, tol=0.001, verbose=False<br>Overall accuracy: **38.17%**								

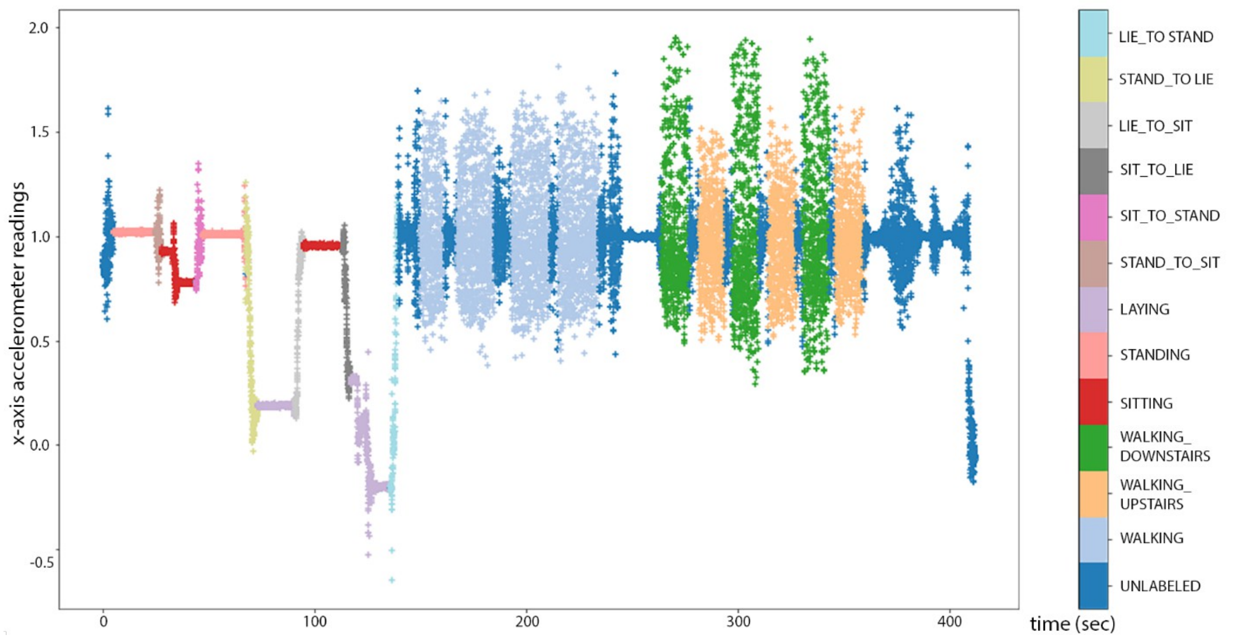

Figure 1A: x-axis accelerometer readings from experiment 1 in the HARuS training dataset.

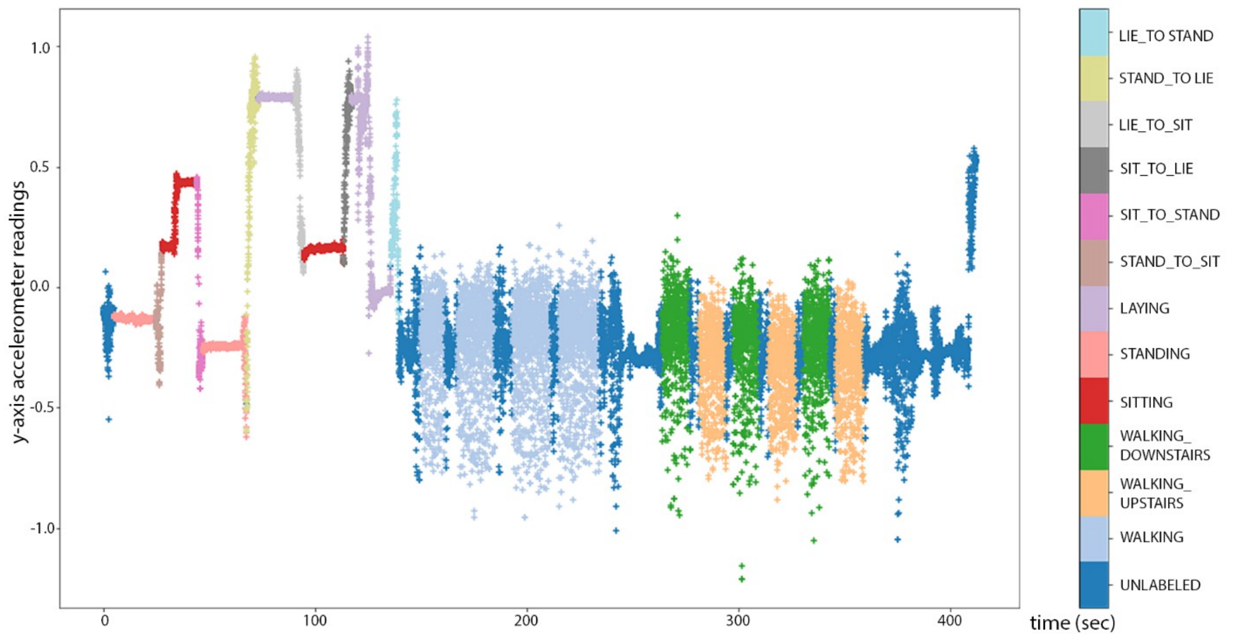

Figure 2A: y-axis accelerometer readings from experiment 1 in the HARuS training dataset.

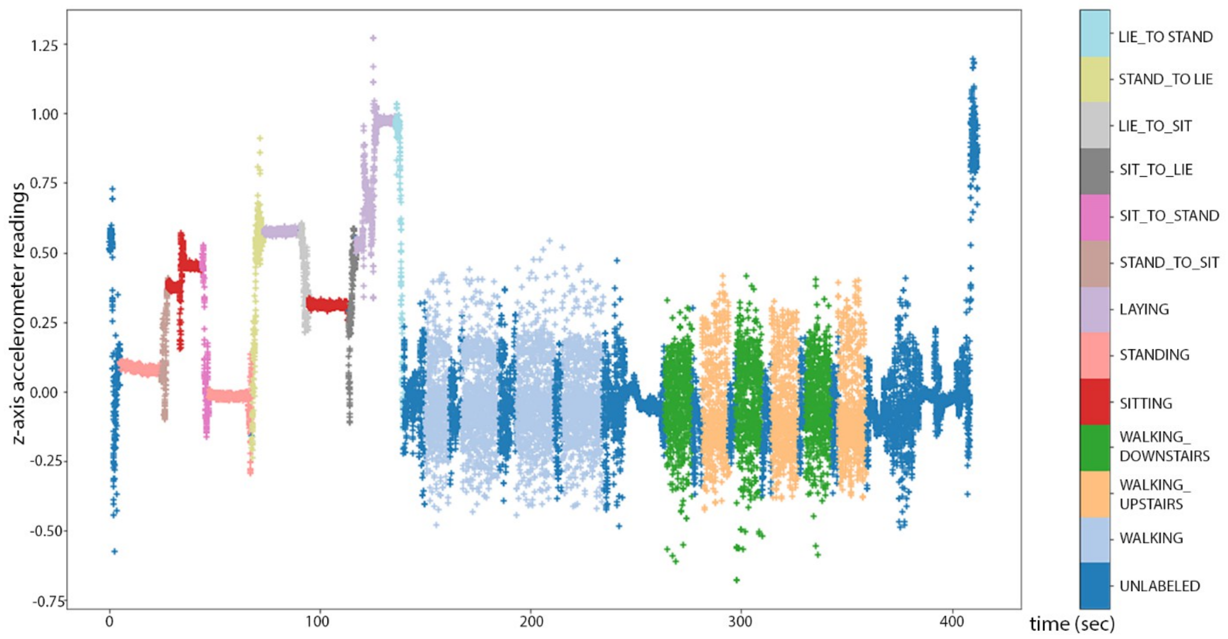

Figure 3A: z-axis accelerometer readings from experiment 1 in the HARuS training dataset.

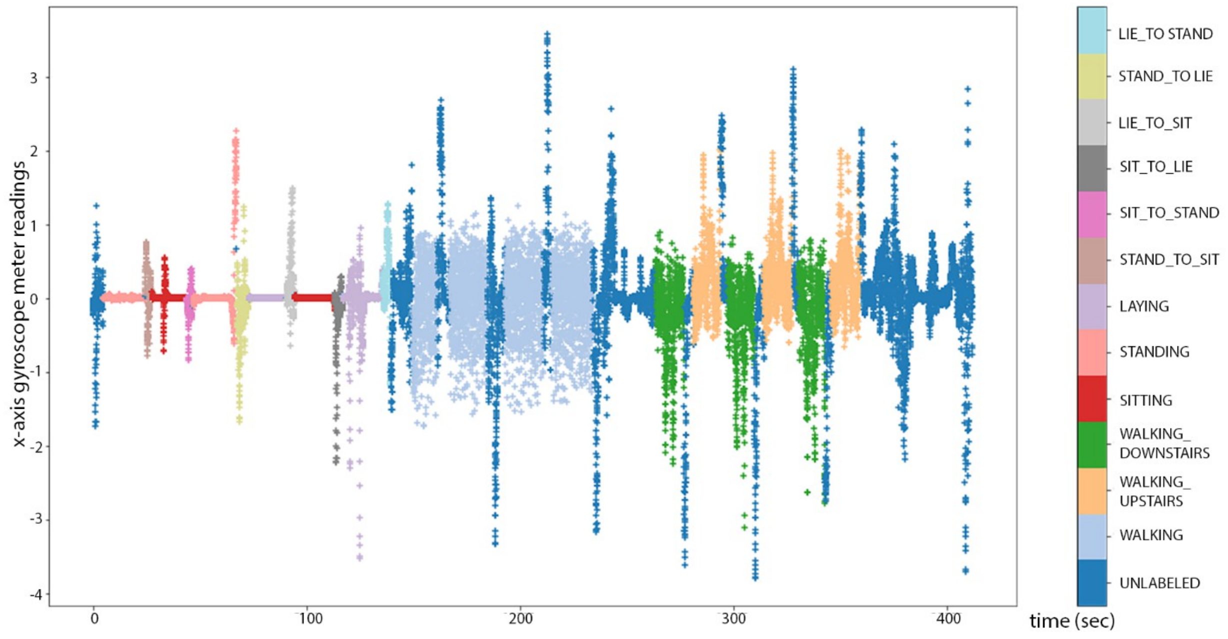

Figure 4A: x-axis gyroscope meter readings from experiment 1 in the HARuS training dataset.

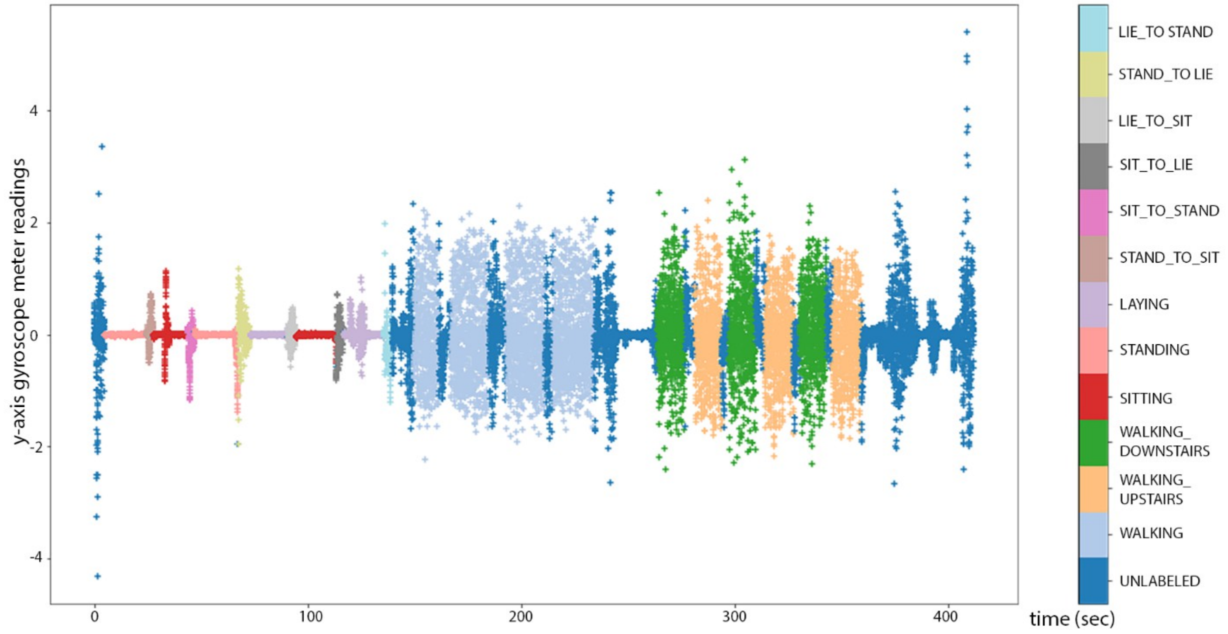

Figure 5A: y-axis gyroscope meter readings from experiment 1 in the HARuS training dataset.

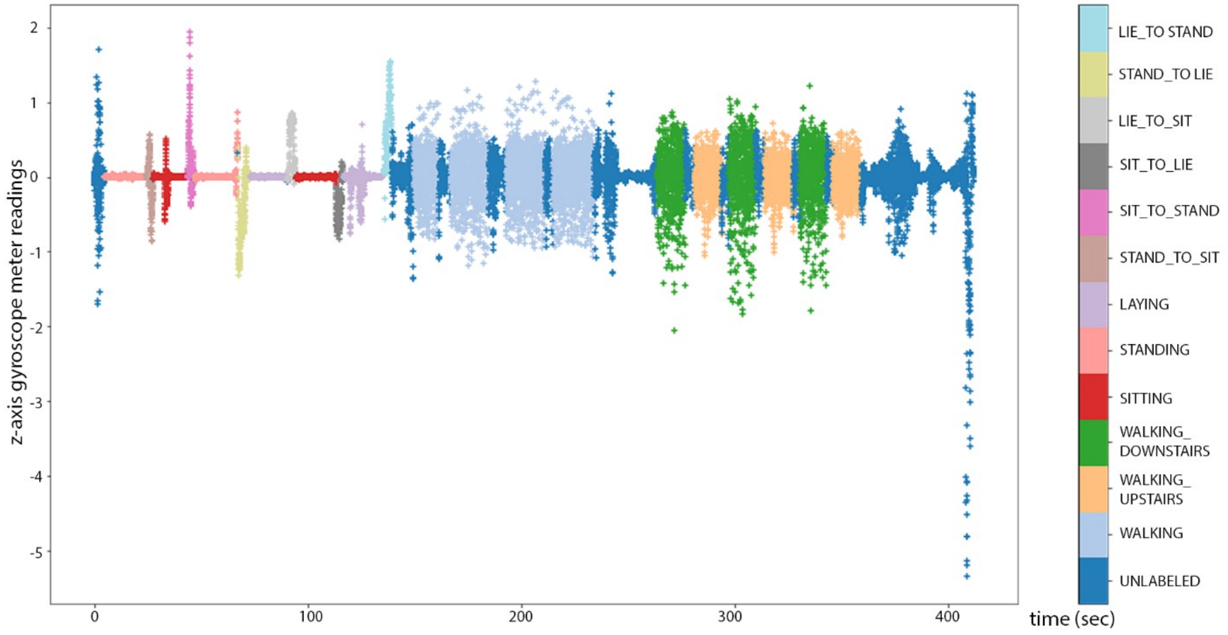

Figure 6A: z-axis gyroscope meter readings from experiment 1 in the HARuS training dataset.

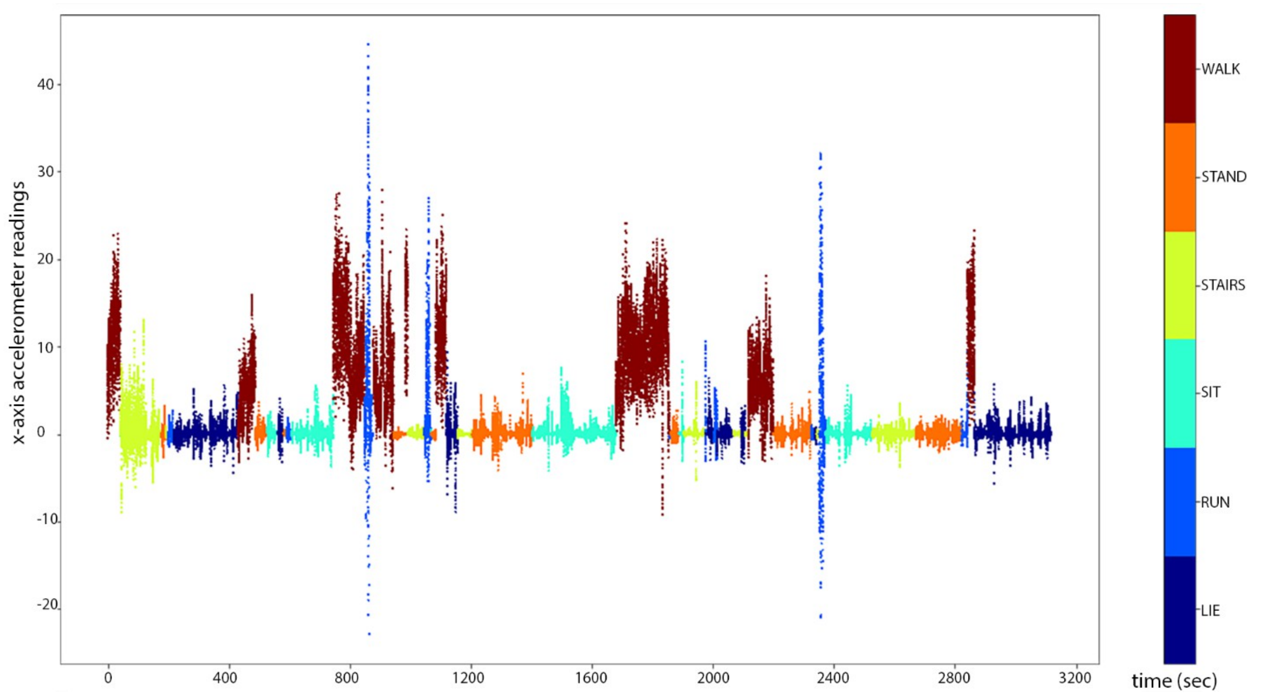

Figure 7A: x-axis accelerometer readings from experiment 1 in the BREATHE training dataset.

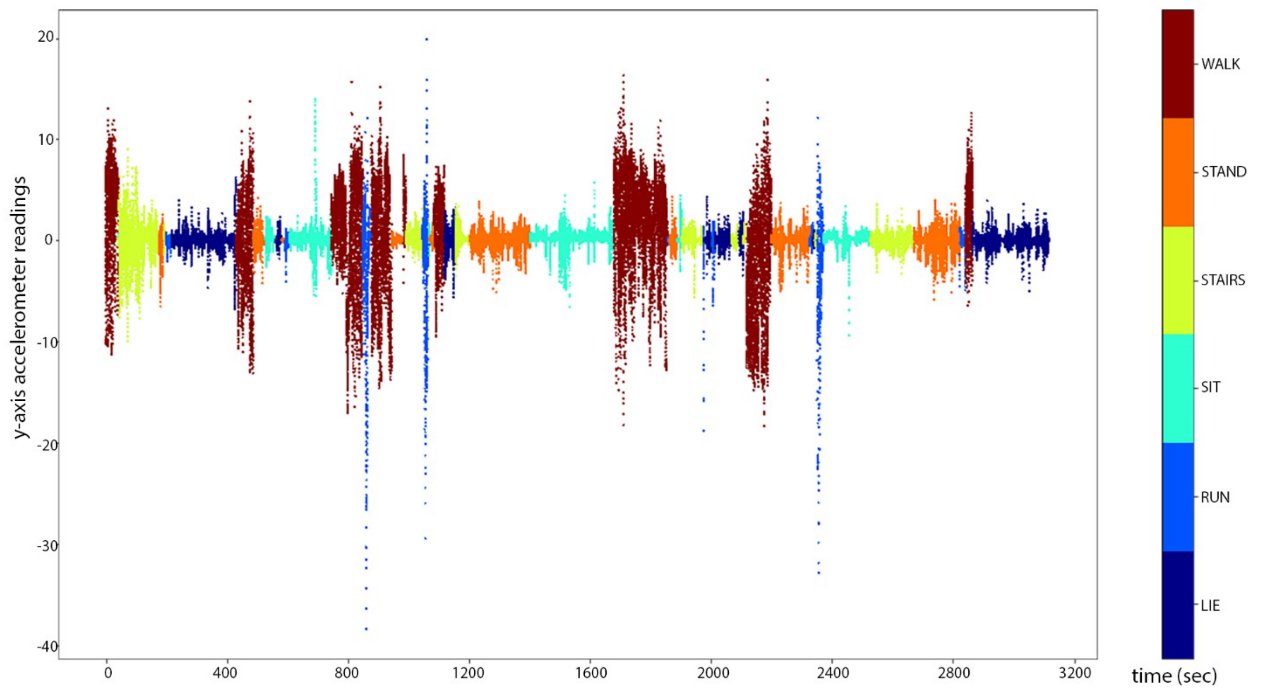

Figure 8A: y-axis accelerometer readings from experiment 1 in the BREATHE training dataset.

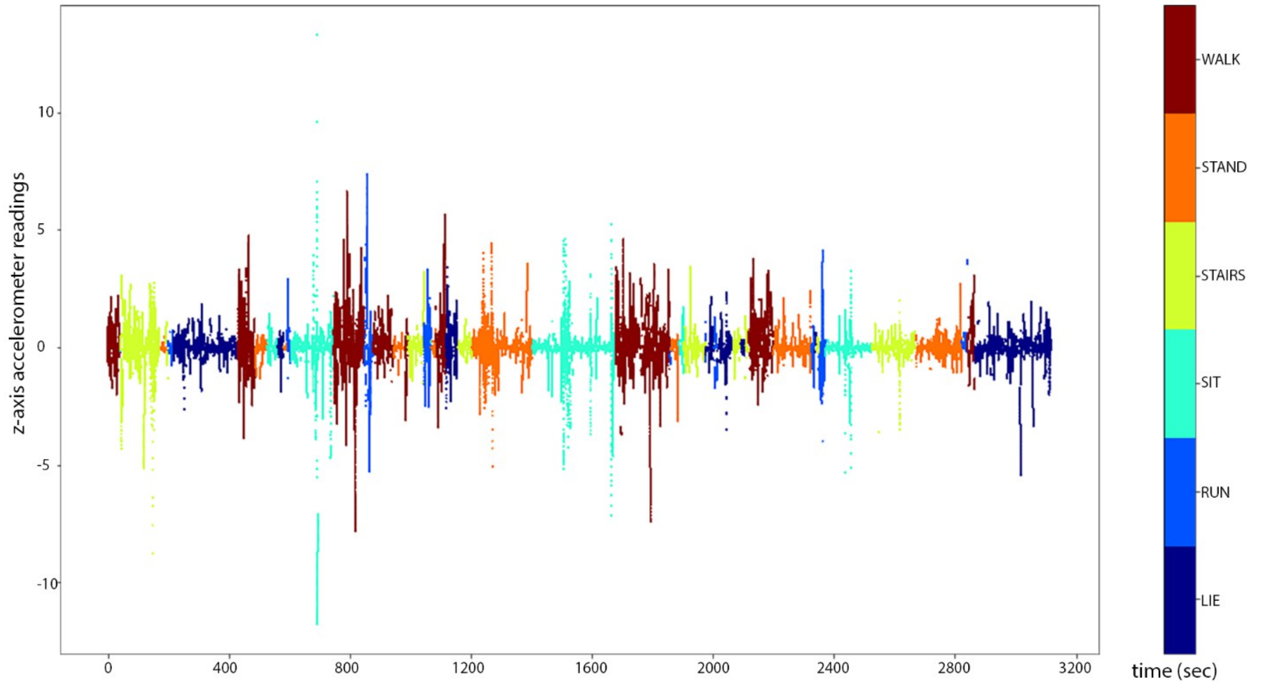

Figure 9A: z-axis accelerometer readings from experiment 1 in the BREATHE training dataset.

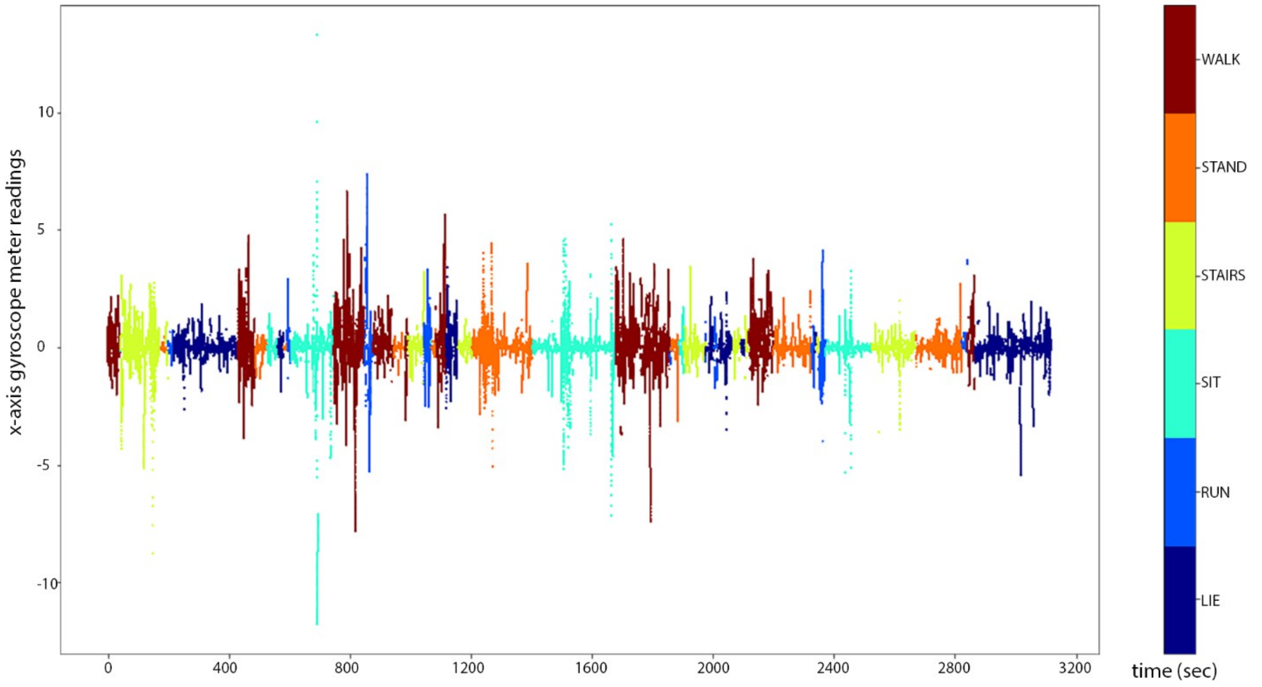

Figure 10A: x-axis gyroscope meter readings from experiment 1 in the BREATHE training dataset.

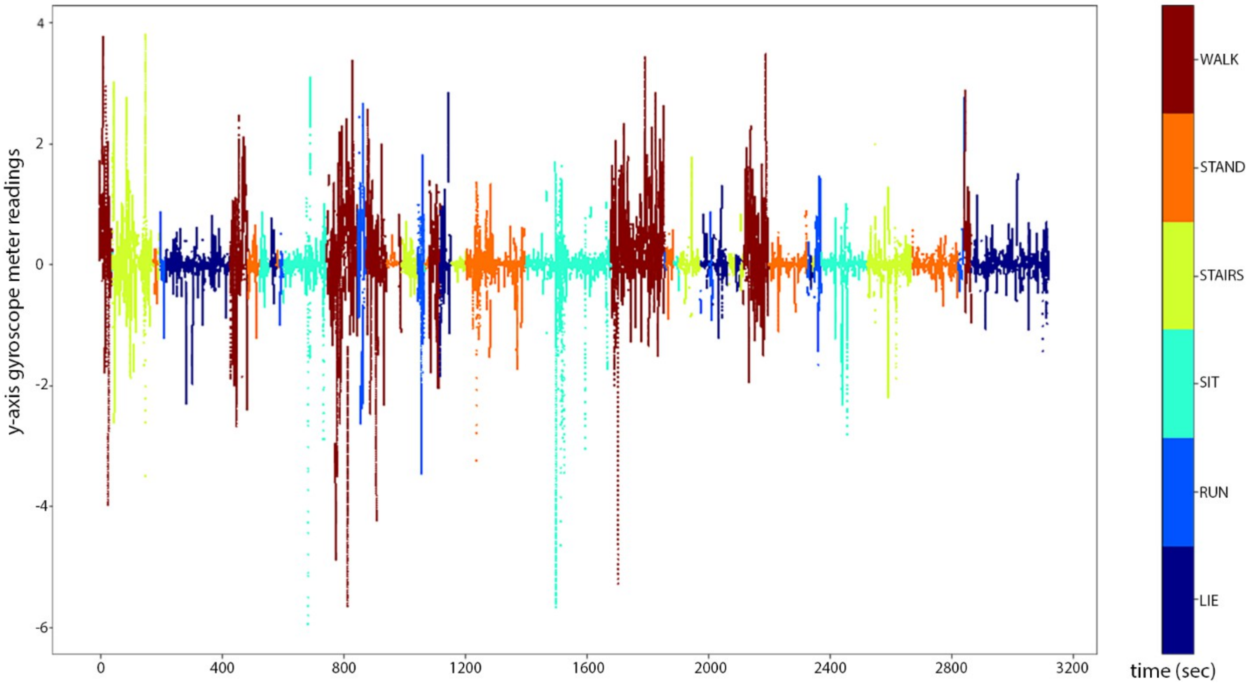

Figure 11A: y-axis gyroscope meter readings from experiment 1 in the BREATHE training dataset.

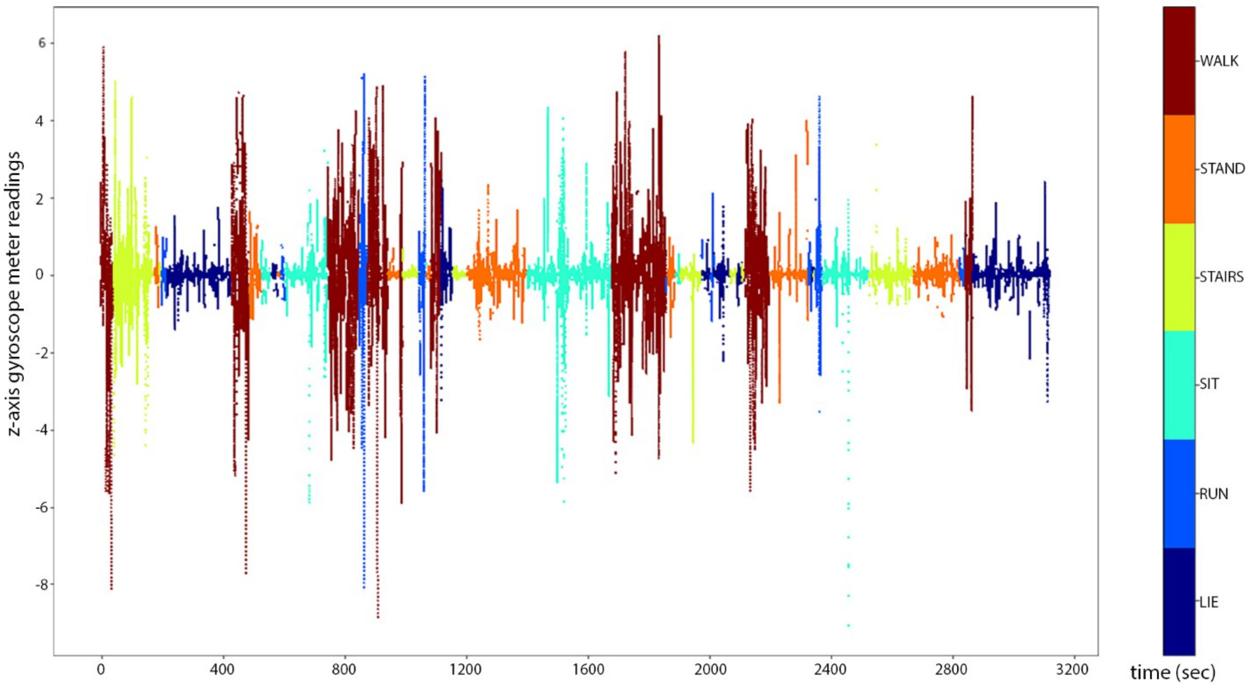

Figure 12A: z-axis gyroscope meter readings from experiment 1 in the BREATHE training dataset.
